# The Interaction of Sub-Monolayer Ta Adatoms and Clusters with Oxygen at the Pt(111) Interface

**DOI:** 10.1021/acs.jpcc.5c00699

**Published:** 2025-03-21

**Authors:** Kevin Bertrang, Tobias Hinke, Sebastian Kaiser, Matthias Knechtges, Federico Loi, Paolo Lacovig, Mirali Jahangirzadeh Varjovi, Friedrich Esch, Alessandro Baraldi, Sergio Tosoni, Aras Kartouzian, Ueli Heiz

**Affiliations:** † TUM School of Natural Sciences, Department of Chemistry, Chair of Physical Chemistry, Technical University of Munich, Catalysis Research Center, Technical University of Munich, Garching D-85748, Germany; ‡ Department of Physics, University of Trieste, 34127 Trieste, Italy; § Elettra-Sincrotrone Trieste, 34149 Trieste, Italy; ∥ Dipartimento di Scienza dei Materiali, Università di Milano Bicocca, 20125 Milano, Italy

## Abstract

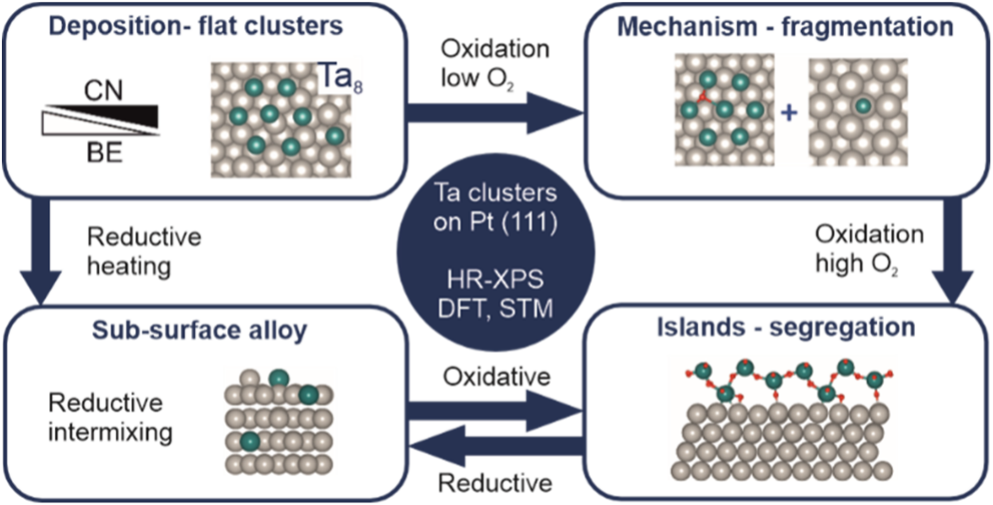

The interaction of submonolayer quantities of size-selected and soft-landed Ta*_n_* (*n* = 4, 5, 6, 8, 13) clusters with Pt(111) is investigated employing high-resolution X-ray photoelectron spectroscopy (HR-XPS), scanning tunneling microscopy (STM), and density functional theory (DFT) simulations. The deposited clusters are monodispersed and stable under ultrahigh vacuum (UHV) conditions at 40 K. They display a size-specific trend in photoemission spectra, which is reasoned in terms of the distinct in plane coordination of Ta atoms in the clusters. Both the Ta coordination number and distance from the Pt surface influence its Bader charge and, accordingly, the oxidation state of the atoms in the Ta cluster. They already fragment in the presence of low amounts of oxygen and form a common oxidation product observed for all cluster sizes. Based on our observations, we propose an oxidation mechanism in the example of Ta_8_ clusters, which is closely comparable to the one discussed in gas-phase studies on the oxidation of cationic Ta clusters of similar size. Concomitant to oxidation-induced fragmentation, the agglomeration into Ta-oxide islands with Ta in an oxidation state of +5 is observed. However, the strong interaction with the Pt surface leads to Ta 4f orbital photoemission features that differ from those commonly observed for Ta_2_O_5_. Computational insights concerning the structure of the Ta-oxide islands indicate flat agglomerates that agree with STM observations. They suggest distinct Ta 4f photoemission contributions from interfacial and surface-related Ta configurations. The respective HR-XPS spectra display specific core-level shifts as a function of bonding configuration and vicinity to the Pt surface. By annealing at 900 K in UHV, we observe oxygen loss and concomitant intermixing of Ta atoms with the Pt subsurface lattice to which results in the formation of a Ta–Pt alloy. These species, Ta-oxide islands, and Ta–Pt alloy, can reversibly interconvert by oxidative surface segregation and reductive intermixing.

## Introduction

Ta and Ta-oxide film-coated Pt components are widely employed in electronic devices, e.g., multilayer ceramic capacitors.^1,2^ Coating the noble metal Pt with a relatively inert Ta-oxide of high dielectricity offers several advantages. The main benefit is increased chemical durability, such as of Ta_2_O_5_-coated Pt electrodes in electrochemical processes.^3−6^ Besides an enhanced performance, new functionalities can emerge from particular arrangements that can be exploited to engineer novel applications, such as Schottky diodes for hydrogen sensing^7^ and memristors for resistive random-access memory applications.^8−12^ In these, (i) the oxidation state of Ta, (ii) the oxygen stoichiometry, and (iii) the migration of constituents are decisive for functionality.^13−15^ Consequently, studying the electronic properties of interfacial structures constituted of Pt, Ta and Ta-oxides can yield fundamental insights into their structure and evolution in different environments, which are of great interest both from a fundamental and applied point of view. The chemical interaction between Ta and Pt in Ta–Pt and Ta–O–Pt interfaces and its evolution during oxidation and film growth remains poorly understood. In particular, an unambiguous attribution of Ta oxidation states remains challenging mainly due to the numerous oxides and suboxides Ta can form and due to the electronic perturbation Ta experiences at the interface to Pt. Thus, attributing a particular oxidation state of Ta to a core-level shift (CLS) observed in X-ray photoelectron spectroscopy (XPS) is often inconclusive, and considerable discrepancies can be found in the literature.^16−19^ These mainly emerge from the predominant technique used to investigate Ta-oxide thin films, sputter-etching combined with XPS.^6,16,18,20−22^ While the method is straightforward and offers valuable insights (both quantitatively and qualitatively) into the chemical composition of a Ta-oxide film, it suffers from limitations due to its invasive nature. The bombardment with high-energy ions (in the kV range) inevitably induces changes in the original depth distribution, and computational reconstruction methods are often required to access information on the unperturbed structure.^23−26^ Hence, pristine structural and chemical information is hardly discernible or not accessible, especially considering the interfacial contributions.

In contrast to sputter-etching, we present a bottom-up study of single Ta atoms and clusters deposited on a Pt(111) single crystal, shedding light on their structural, electronic, and chemical characteristics while emphasizing the interfacial constitution. We investigate the behavior of metallic Ta clusters at the Pt(111) surface and their reaction with oxygen at low (40 K) and elevated (900 K) temperatures using high-resolution X-ray photoelectron spectroscopy (HR-XPS) complemented by density functional theory (DFT) simulations and scanning tunneling microscopy (STM). Hereby, the oxidation of Ta atoms and clusters is monitored upon increasing exposure to O_2_, elucidating the oxidation products.

In previous work, we recently investigated the interaction of evaporated Ta atoms with the Pt(111) surface at 40 K.^27^ Different surface adsorption sites for Ta adatoms were identified, with preferred adsorption to step edges. Summarizing this article, we found that the transient mobility of the evaporated atoms, with a residual kinetic energy of 0.28 eV/atom, promotes the formation of small Ta agglomerates. At elevated temperatures (900 K) in ultrahigh vacuum (UHV), Ta diffusion into the Pt surface is enabled (intermixing), whereby a subsurface alloy forms in which Ta accumulates in the second Pt surface layer, while bulk diffusion is not observed. Both adsorbed Ta adatoms on the Pt surface, and substitutional Ta in the Pt subsurface layer strongly interact with Pt and display a positive charge (+2). Hence, Ta atoms oxidize in contact with Pt, which is reflected in a substantial CLS to higher binding energy (BE) (1.5–2.0 eV) comparable to that of undercoordinated Ta atoms of exposed Ta single-crystal surfaces.^28^ This alloy can be oxidized in an oxygen atmosphere at elevated temperatures (900 K) to yield Ta-oxide islands (surface segregation). Ta-oxide islands and Ta–Pt alloy can be converted into one another by reductive intermixing and oxidative segregation of Ta.

In this article, we extend our study to size-selected supported Ta clusters, elucidating the stability of Ta*_n_* (*n* = 4, 5, 6, 8, 13) clusters on Pt(111) in the presence and absence of oxygen. Performing the HR-XPS measurements at 40 K restricts surface diffusion of the monitored Ta species and strongly reduces phonon-induced broadening of the core-level spectra. Coupled with the overall energy resolution (better than 50 meV), fundamental spectroscopic insights can be derived from the rich and complex Ta 4f spectra of supported Ta clusters. Furthermore, we can exploit oxidation through atomic oxygen. This method^29^ ensures access to the highest oxidation state, which is of particular interest concerning the observed BEs for Ta-oxides reported in our previous work,^27^ indicating an oxidation state lower than Ta^5+^. Based on the combined HR-XPS and DFT results, an oxidation mechanism is proposed for Ta_8_ clusters, providing structural and electronic insights into the formation of the resulting oxidation product common to all cluster sizes. The product of oxidation is further characterized by room-temperature STM measurements in which the comparability to the low-temperature core-level measurements is reasoned in terms of the high similarity of the XPS spectra after oxidation at both low and high temperatures. Finally, and conclusive with our findings on Ta single atoms, a reductive intermixing of the oxidation product and subsequent oxidative surface segregation is observed.

## Experimental Section

### Sample Preparation

All XPS experiments were performed at the SuperESCA beamline of the Elettra synchrotron radiation facility (Trieste, Italy) in a UHV chamber at a 1 × 10^–10^ mbar base pressure. A Pt(111) single crystal was prepared by subsequent cycles of sputtering with Ar^+^-ions (*E*_kin_ = 1.5 keV, *p*_Ar_ = 3.6 × 10^–6^ mbar, *I* = 10 μA, 300 K, 20 min) followed by annealing >900 K for 1 min to ensure surface reconstruction (*T*_Hüttig_ = 608 K).^30^ Carbon impurities were removed by annealing in an O_2_ atmosphere (*p*_O_2__ = 1 × 10^–7^ mbar, 870 K, 5 min), followed by the removal of residual adsorbed oxygen in H_2_ atmosphere (*p*_H_2__ = 5 × 10^–8^ mbar, 670 K, 5 min). The process was repeated until no more contaminations were identified in the O 1s, C 1s, Ta 4f, and Pt 4f regions. The heating of the sample is enabled by electron bombardment from resistively heated W-filaments placed close to the back of the sample. The cluster source setup ENAC (Exact Number of Atoms in each Cluster) developed by the Nanoscale Materials Laboratory of the Department of Physics of the University of Trieste and Elettra Sinctrotrone Trieste, which has proven very successful for similar cluster studies,^29,31−34^ was employed for cluster generation. The source and the ion optics for charge and mass selection are based on the design of Heiz et al. and are described in detail elsewhere.^35^ For the purpose of the following experiments, the cluster source was connected to the preparation chamber of the SuperESCA beamline, where sample cleaning and cluster deposition were performed. A valve gate separates the latter chamber from the analysis chamber, where the HR-XPS measurements of the as-deposited Ta species are conducted. Thus, sample preparation and analysis are carried out in situ. Furthermore, the chamber is connected to a gas manifold, enabling the exposure of the sample to reactive gases while monitoring changes in the photoemission spectra. Atomically precise Ta clusters of distinct sizes were deposited on a Pt(111) crystal at 40 K. The following cluster coverages per ML of Pt (ML_Pt_) were used: Θ(Ta_4_) = 0.10% clusters/ML_Pt_, Θ(Ta_5_) = 0.13% clusters/ML_Pt_, Θ(Ta_6_) = 0.11% clusters/ML_Pt_, Θ(Ta_8_) = 0.10% clusters/ML_Pt_, Θ(Ta_13_) = 0.06% clusters/ML_Pt_ with respect to the atom density of the Pt(111) surface which corresponds to 1.5 × 10^15^ atoms/cm^2^. Before deposition, the substrate was flash annealed up to 500 K to remove adsorbates accumulated from the chamber residual gas background. Ta atoms (Θ(Ta_1_) ≈ 2% atoms/ML_Pt_) were evaporated on the Pt(111) surface from a Ta wire (thickness: 0.125 mm, purity: 99.9%, Goodfellow) as described elsewhere.^27^

All STM measurements were performed in a different UHV chamber at 1 × 10^–10^ mbar base pressure at the Technical University of Munich. The Pt(111) single crystal was prepared by cycles of sputtering with Ar^+^-ions (*E*_kin_ = 1 keV, *p*_Ar_ = 4.0 × 10^–5^ mbar, *I* = 10 μA, 300 K, 20 min), followed by annealing >900 K for 5 min. Subsequently, the crystal was annealed at 720 K in an O_2_ background (*p*_O_2__ = 5 × 10^–7^ mbar, 10 min) and ramped to 1000 K in UHV. Similarly to the other experiments, the Ta clusters were deposited with a cluster generation setup based on the design of Heiz et al.^35^ under soft-landing conditions at room temperature. A Ta_4_ coverage of 0.13% clusters/ML_Pt_ and a Ta_8_ coverage of 0.26% clusters/ML_Pt_ have been obtained.

### Oxidation

**Figure 1:**
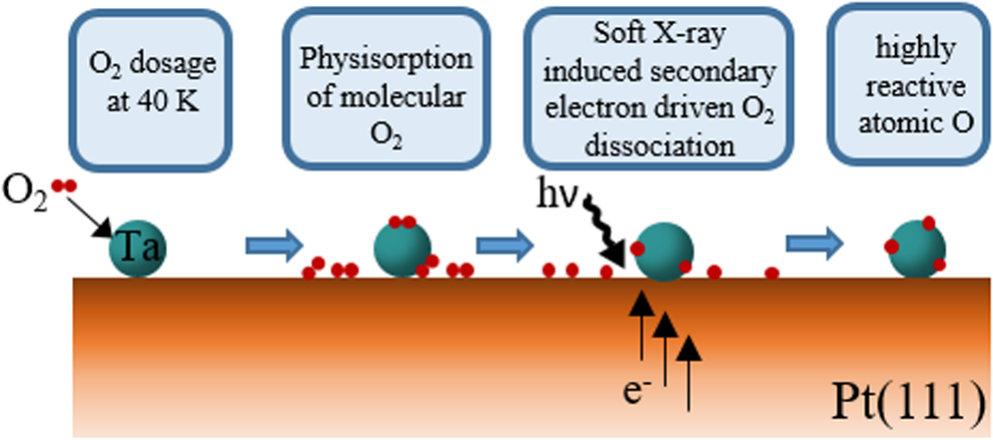
Scheme of the low-temperature oxidation procedure using physisorbed molecular O_2_. The soft X-ray radiation causes the release of secondary electrons that lead to the dissociation of O_2_, which forms highly reactive atomic oxygen.

The oxidation of evaporated Ta atoms and deposited Ta clusters in the HR-XPS measurements at 40 K was performed according to a previously reported procedure^29,31^ schematically depicted in Figure 1. Oxygen was predosed at 40 K (0.1–10 L), yielding a physisorbed layer of molecular O_2_. Irradiation of the sample with soft X-rays leads to the release of secondary electrons from the substrate that induce dissociation of the physisorbed O_2_ molecules to form highly reactive atomic oxygen, which permits reaching the highest accessible oxidation state of the adsorbed Ta particles. Oxidation at elevated temperature (900 K) was performed in a background of molecular O_2_ (*p*_O_2__ = 1 × 10^–6^ mbar) instead. The transformation of the Ta-oxide islands into the Ta–Pt alloy was achieved by UHV annealing at 900 K until no further changes in the photoemission spectra were distinguished (<10 min). The cluster oxidation in the STM experiments was performed in a background of molecular O_2_ (*p*_O_2__ = 1.2 × 10^–7^ mbar, 15 min) at room temperature. Thereafter, the sample was annealed at 600 K for 15 min in UHV.

### HR-XPS Measurements

In the photon energy range applied here for the Ta 4f and Pt 4f spectra, a resolution better than 50 meV can be achieved.^36^ Photoelectrons were collected and filtered using a Phoibos 150 mm mean-radius hemispherical electron energy analyzer (SPECS, Germany) in fixed analyzer transmission mode and detected with a 1D delay line detector. The following set of orbitals was probed at the corresponding photon energies indicated in brackets: O 1s (*h*ν = 650 eV), C 1s (*h*ν = 400 eV), Pt 4f (*h*ν = 220 eV), and Ta 4f (*h*ν = 150 eV). All acquired spectra were measured in normal emission geometry. The analyzer was operated in fixed analyzer transmission at a pass energy of 5 eV. Spectra were normalized to the photon flux of the incident beam and the number of scans. The core–electron binding energies of the experimental spectra were referenced to the Fermi energy, measured subsequently and under the same conditions. To compare the computational core–electron BEs to the experimental ones, they have been aligned using the Ta–Pt subsurface alloy formed at 900 K in UHV as an internal experimental reference for Ta (IER_Ta_). This alloy species appears in the experiment with a well-defined peak at a BE of 23.59 eV, observed under the same measurement conditions as all the other Ta species discussed in this article.^27^ The calculations are then referenced by taking the calculated BE of the defect-free Ta(110) slab (19.35 eV) and the Ta 4f CLS of the calculated subsurface alloy (+1.47 eV) with respect to the slab for realigning all calculated Ta 4f BEs. This leads to a realigned calculated BE for the Ta(110) photoemission of 22.12 eV, which is in agreement with experimental literature values.^28^ Similarly, the calculated Pt 4f BEs of surface atoms in Pt(111) can be calibrated according to the experimental value of 70.52 eV corresponding to the surface component in Pt(111).^37^

### STM Measurements

The STM images were acquired in constant current mode with a commercial Scienta Omicron VT-AFM instrument, using homemade etched W tips. The images were processed with Gwyddion using plane subtraction and row-by-row alignment tools for background correction.^38^ The height distribution of the particles was determined using a home-written Igor Pro Wavemetrics routine by detecting the particles via an intensity threshold, drawing a profile through the cluster maximum, and determining the cluster height with respect to the median background of the image.

### Computational Details

All calculations were performed with the code VASP6.^39,40^ The interaction between the core electrons and the nuclei is described with the PAW formalism.^41,42^ The plane-wave basis set was expanded up to a kinetic energy of 400 eV. Truncation criteria of 10^–5^ eV for the electronic loop and 0.01 eV/Å for the ionic loop have been set. A PBE exchange-correlation functional was adopted.^43^ The core-level energies have been calculated within the initial state approximation and realigned to the experimental BEs using the pure Ta(110) surface as a common reference. This approach is often found to yield a reasonable agreement with experimental findings for both metal adlayers and bulk alloys^44^ and is adequate in particular when oxidation state changes lead to BE differences up to several eV. A 5 × 5 Pt(111) cell has been adopted to accommodate the Ta_4_ clusters, while an 8 × 8 cell has been adopted for Ta_8_. The reciprocal space has been sampled with a Monkhorst net of 4 × 4 × 1 and 2 × 2 × 1 *K*-points, respectively. Pt(111) has been modeled with a five-layer slab, where the atoms from the bottom layer are frozen in their bulk positions while all other atoms are free to relax. An empty space of at least 20 Å is included in all supercells to avoid spurious interactions with the replica. The adsorption energy per Ta atom of Ta*_n_* species on Pt(111) has been calculated as follows
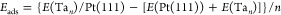
where Ta*_n_*/Pt(111) refers to a Ta*_n_* species bound to Pt, Pt(111) is the clean Pt surface, and Ta*_n_* is the gas-phase tantalum cluster in its most stable geometry and electronic ground state.

The Ta-oxide islands were modeled recurring to a two-dimensional tantalum oxide slab supported on Pt(111). The D3 Grimme parameterization and the Becke–Johnson damping function were adopted to account for the metal-oxide noncovalent interactions.^45,46^ We focused on the β-Ta_2_O_5_ phase, whose most stable surface is the (100), as assessed in a previous computational work.^47^ However, due to the very poor lattice match between Ta_2_O_5_(100) and Pt(111), the interface with Pt(111) was simulated by cutting the oxide along another, less stable, low-index surface, namely (001). A model with a minor lattice strain (+1.7%, +1.3%) released on the oxide film was then constructed with a (3 × 4) Ta_2_O_5_(001) on a 4 × √3 Pt(111) coincidence lattice.

## Results and Discussion

### Ta Clusters

**Figure 2:**
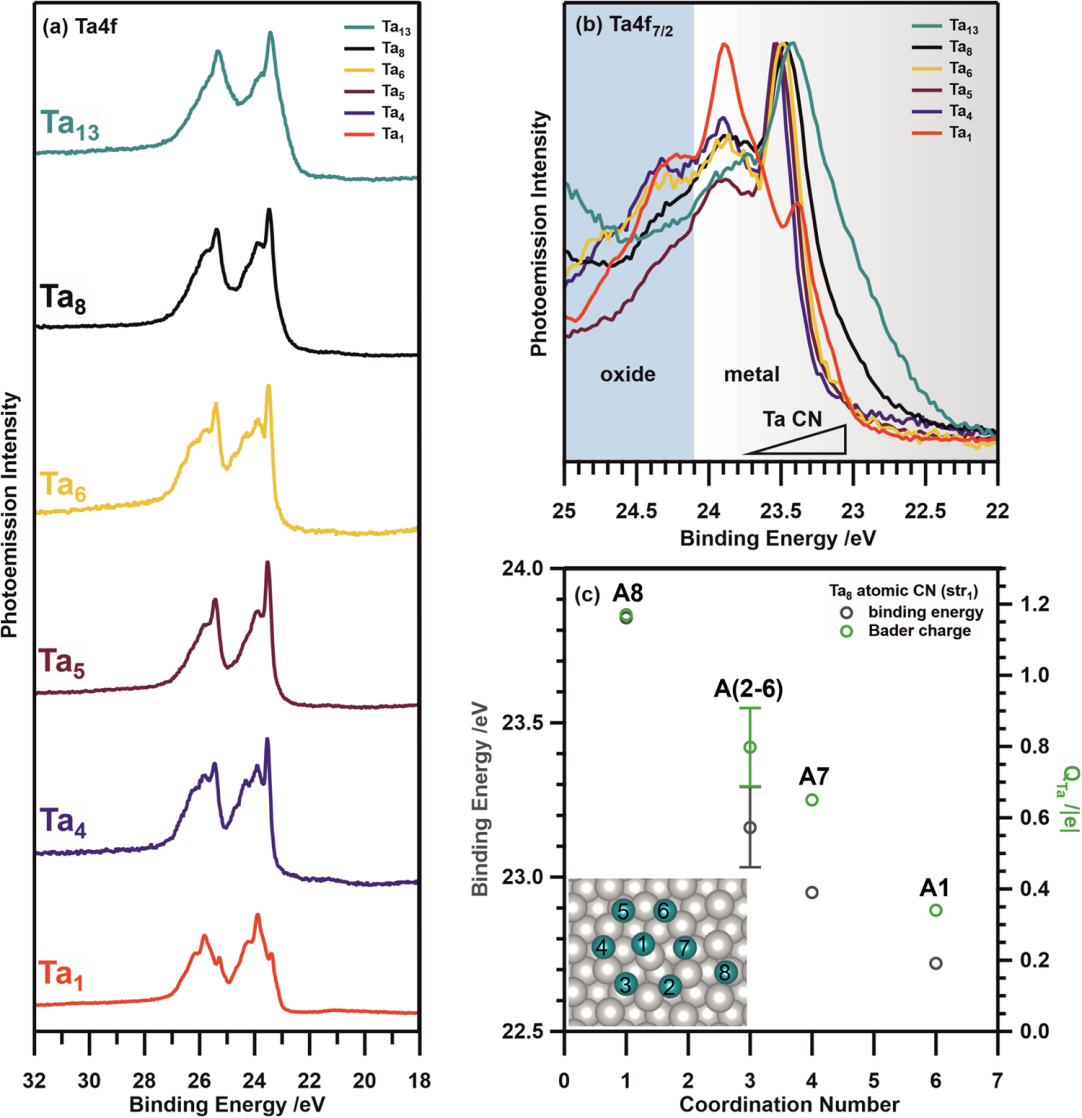
(a) Stacked overview of the Ta 4f XPS spectra (*h*υ = 150 eV) of Ta atoms directly after evaporation and Ta*_n_* clusters (*n* = 4, 5, 6, 8, 13) directly after deposition on Pt(111) at 40 K. From bottom to top are displayed: Ta adatoms (red), Ta_4_ (dark blue), Ta_5_ (brown), Ta_6_ (yellow), Ta_8_ (black) and Ta_13_ (turquoise). All Ta peaks are found to be considerably shifted toward higher BE with respect to metallic Ta^0^ (21.64 eV).^28,48,49^ (b) Cut-out of the Ta 4f_7/2_ region for Ta*_n_* clusters (*n* = 4, 5, 6, 8, 13) after the spectra are normalized to their maximum. The shadings indicate two main BE regions related to metallic (gray gradient) and oxidic (blue) Ta contributions. The gradient over the metallic region reflects the change in BE as a function of coordination within the Ta clusters as discussed in the main text. (c) Simulated BEs (green) and Bader charges (gray) of the Ta atoms (A1–A8) in a Ta_8_ cluster deposited on Pt(111) (see inset) depending on the CN of the atoms. Atoms A(2–6) represent an average of atoms with the same CN.

Ta_n_ clusters (*n* = 4, 5, 6, 8, 13) were deposited in UHV, under soft-landing conditions (*E*_kin_ < 1 eV/atom), on a Pt(111) single crystal at 40 K using the laser ablation cluster generation setup ENAC.^29^ The corresponding 4f core-level spectra of the as-deposited Ta*_n_* cluster sizes are shown in Figure 2a as a stacked overview and in Figure 2b as normalized to their maximum intensity. Duplets with a characteristic spin–orbit coupling of 1.91 eV and the 4:3 spin multiplicity intensity ratio (corresponding to Ta 4f_7/2_ and Ta 4f_5/2_) are observed. The Ta 4f_7/2_ peaks appear in the range 22–25 eV, where they can overlap with O 2s photoemission signals. All observed Ta 4f emissions show a considerable shift to higher BEs with respect to emissions commonly observed for metallic Ta^0^ bulk^28,48,49^ (21.64 eV) and surfaces, e.g., Ta(100)^48^ (22.39 eV), Ta(110)^28^ (21.97 eV) and Ta(111)^49^ (22.04 eV). This is similar to the CLSs found for Ta adatoms on the same support, where the interaction between the early transition metal Ta and the noble metal Pt leads to a formal oxidation of Ta by Pt. Ta adatoms are found to display an oxidation state of approximately +2, as suggested by simulations.^27^

Strong spectral similarities are found for all investigated Ta cluster sizes. Common photoemission regions are identified as indicated by the two shaded areas in Figure 2b. They can be attributed to contributions from metallic Ta clusters (gray gradient), ranging from 22.0 to 24.1 eV and oxidic Ta species (blue), with a Ta 4f_7/2_ BE > 24.1 eV. Overall, the photoemission peaks from metallic Ta clusters found below 23.6 eV are most pronounced and exhibit a size-specific trend: For smaller clusters, e.g., Ta_4_, a relatively sharp photoemission onset is observed at ∼23 eV. In contrast, this onset gradually evolves for larger clusters (investigated up to Ta_13_), as observed in the contributions at the low BE end of the spectra. These photoemission features are in good agreement with those observed for Ta agglomerates (23.4 eV), which have been previously identified after deposition of Ta atoms. The formation of agglomerates was assigned to the transient atom mobility due to residual *E*_kin_ of ∼0.28 eV/atom upon evaporation.^27^ Comparison of the Ta cluster with the Ta adatom (red) spectra indicates a contribution similar to that observed for Ta adatoms (23.6–24.1 eV) and is present in the spectra of all cluster sizes. To further investigate the 4f_7/2_ spectral trend observed for different Ta cluster sizes and the origin of contributions similar to Ta adatoms emissions to the cluster spectra, insights into cluster geometry and stability are required. Therefore, the structure and stability of Ta_4_ and Ta_8_ on Pt(111) have been investigated by DFT and STM.

**Figure 3:**
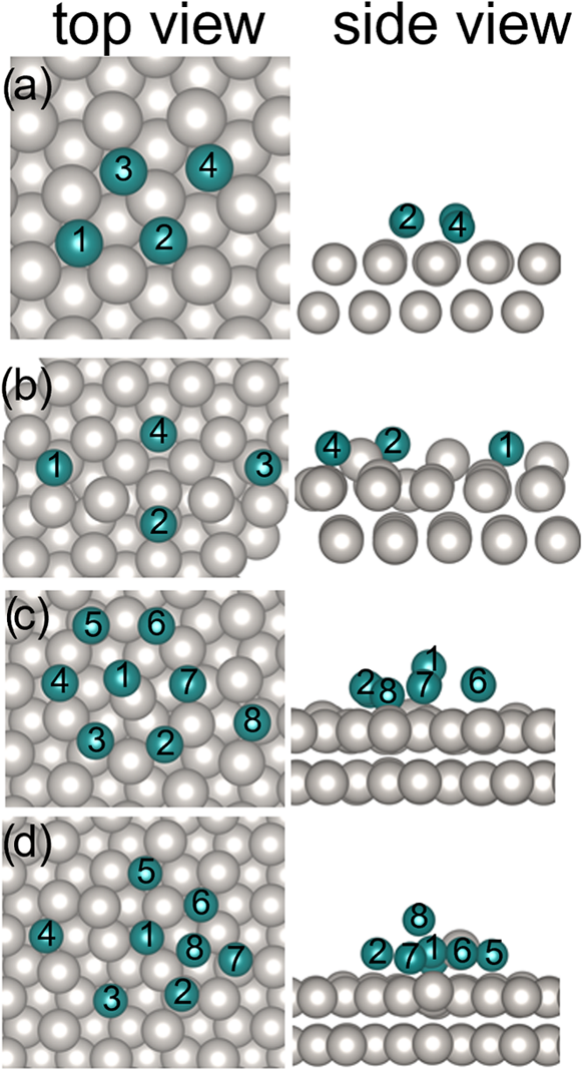
Computed Ta_4_ and Ta_8_ structures on Pt(111). Top views (left column) and the respective side views (right column) of the structures str_1_(Ta_4_) (a), str_2_(Ta_4_) (b), str_1_(Ta_8_) (c), and str_2_(Ta_8_) (d).

For Ta_4_ clusters, a gas-phase simulation returns a tetrahedral shape, see Figure S1 left, and a closed-shell ground state configuration.^50^ The adsorption energy (*E*_ads_) per Ta atom, however, reveals that a flat isomer, str_1_(Ta_4_), is favored (*E*_ads_ = −3.25 eV/atom) on the Pt(111) support, Figure 3a, and tetrahedral structures are much less stable. Two of the Ta atoms (A1, A4) of the flat tetramer are in good registry with the underlying hollow sites and display a core–electron BE of 23.43 eV, see Table S1, slightly smaller than that of Ta adatoms on the surface. The remaining two atoms (A2, A3) display a poorer registry and a smaller BE of 22.93 eV. Further energy is gained if one Ta atom takes over for Pt in a lattice site: str_2_(Ta_4_) has an *E*_ads_ of −3.96 eV/atom, see Figure 3b. However, given the low temperature of the experiments this rearrangement will likely be experimentally inaccessible. The Ta–Pt substitution that contributes to the overall stabilization is supposedly hindered by an energy barrier that is hard to account for in the calculations. STM measurements on the Ta_4_ cluster on Pt(111) (0.13% clusters/ML_Pt_, 293 K), see Figure S2, reveal that, indeed, a flat geometry is adopted on Pt(111), and the height profile indicates an apparent height for Ta_4_ clusters of ∼0.12 nm. While intact, monodisperse Ta_4_ clusters dominate, some particles accumulated at step edges and sintered ones on the terraces are also observed. Furthermore, some fragments of unspecified size can be found. In this context, it is interesting to point out that the *E*_ads_ for Ta_4_ is directly comparable to the case of Ta adatoms. A crosscheck immediately reveals that a fully dispersed arrangement of atomic Ta species on the surface (*E*_ads_ = −3.84 eV/atom, Table S1) is of comparable stability to that of the flat Ta_4_ structure. Thus, in the case of Ta_4_, the thermodynamic drive toward dispersion seems to be kinetically accessible at room temperature, as indicated by the fragments observed in the STM images. Nevertheless, at 40 K and under irradiation, no evidence for X-ray beam damage-induced fragmentation is found, neither for Ta_4_ clusters nor any other investigated cluster size, as shown in Figure S3. Hence, the origin of atomic contributions in cluster XPS spectra at 40 K cannot be explained by probe-induced fragmentation and indicates a different origin.

In the gas-phase, Ta_8_ assumes the shape of a capped octahedron;^50^ see Figure S1 right. On the Pt(111) surface, however, our calculations show that this arrangement is not stable upon adsorption, and the Ta_8_ cluster again, similar to Ta_4_, tends to spread on the surface, maximizing the interaction with Pt. In particular, two minima with different structures have been identified, namely str_1_(Ta_8_) and str_2_(Ta_8_), which are displayed in Figure 3c,d as top and side views, respectively. Str_1_(Ta_8_) appears as an almost flat structure, with a maximum height of 0.29 nm with respect to the Pt apical plane. Six Ta atoms (A2–A7) occupy bridge Pt–Pt sites in an almost hexagonal arrangement; one Ta atom is hosted in a Pt_3_-hollow side in the middle of the hexagon (A1), and the last one is adsorbed aside on a hollow position (A8). The *E*_ads_ = −2.13 eV/atom, thus indicates a substantial thermodynamic gain related to the adsorption and almost complete flattening of the Ta_8_ cluster on Pt(111). The calculated Ta 4f BEs span from 22.72–23.84 eV, see Table S2. The corresponding Bader charges and interatomic Ta–Ta distances are reported in Tables S2 and S3, respectively.

Similarly to the case of Ta_4_, an even more stable arrangement, str_2_(Ta_8_), could be identified and is shown in Figure 3d: Interestingly, during the structure relaxation, spontaneous incorporation of a Ta atom (A4) into Pt(111) took place, and the expelled Pt atom remained aggregated to the Ta cluster. In this case, the adsorption energy is −2.68 eV/atom. Str_2_(Ta_8_), thus, is in principle more stable than str_1_(Ta_8_). In our previous work,^27^ we showed how the Ta–Pt substitution is thermodynamically favorable in surface sites, and an even stronger stabilization is envisaged if the Ta impurity migrates to a bulk Pt site, in agreement with a previous computational work.^51^ However, like for Ta_4_, Ta–Pt substitution to form str_1_(Ta_8_) is probably hindered at 40 K. The alloying with Pt induces a general increase in the calculated Ta 4f BEs, see Table S1. Notably, both structures discussed here display a quasi-flat arrangement of the Ta atoms, compatible with apparent heights typical of adsorbed monolayer structures.

**Figure 4:**
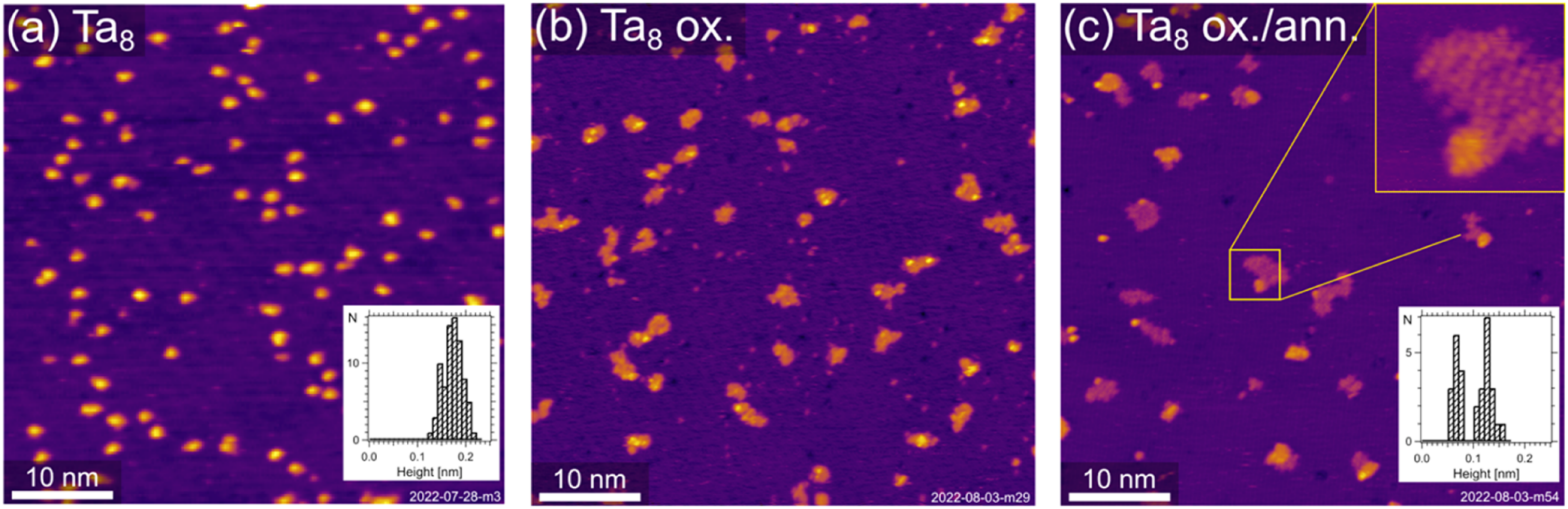
STM images of Ta_8_ clusters on Pt(111) illustrate the morphology of deposited clusters and their changes upon oxidation and/or annealing. The clusters were deposited and measured at room temperature (0.26% clusters/ML_Pt_). (a) Display of the as-deposited pristine Ta_8_ clusters. The height histogram (inset) reveals the monodisperse formation of clusters with an apparent height of 0.18 nm. (b) After oxidation at room temperature (*p*_O_2__ = 1.2 × 10^–7^ mbar, 15 min), the clusters have considerably sintered to form larger, flatter oxidic species (0.1% species/ML_Pt_), on which occasionally small, higher features reside. (c) After further annealing (*p*_O_2__ = 1.2 × 10^–7^ mbar, 600 K, 15 min), which also leads to the partial desorption of oxygen from the Pt surface, the image resolution slightly improves (see, e.g., the enlarged inset). The dominant flat terraces now have an apparent height of 0.07 nm, while small second-layer areas appear at a height of 0.13 nm (see histogram inset). Imaging parameters for all images: *U*_bias_ = 1.5 V, *I*_t_ = 0.3 nA, 50 × 50 nm^2^.

The STM measurements of Ta_8_ clusters, deposited and recorded at room temperature, on Pt(111) in Figure 4a display monodisperse particles with a mostly flat two-dimensional (2D) geometry. The height histogram sampled over a terrace (inset of Figure 4a) indicates an apparent cluster height of ∼0.18 nm, which is smaller than an atomic step height of a Pt(111) surface (0.226 nm).^52^ This lower apparent height of the Ta clusters could be related to different electron densities of states of the two involved metals that considerably affect the observed heights in the STM topography of heteroepitactic contacts. The clusters are found intact (over several hours) with no conclusive indication of the presence of Ta fragments (<Ta_8_) or Ta adatoms on the Pt surface, even at room temperature. Additionally, under X-ray irradiation (150 eV), negligible changes are observed over a series of five XPS spectra (∼8 min), see Figure S3. Hence, considering the sequence of spectra and STM, atomically precise Ta_8_ clusters are stable upon deposition at *T* < 293 K and under irradiation at 40 K.

Overall, considering the flat structure of Ta_4_ and Ta_8_, as well as the common trend of a preferred 2D geometry for metal clusters on metal supports^53^ to minimize surface free energy, such as observed for Ag_19_ on Pt(111)^54^ or Pd_19_ on Rh(111),^55^ a flat geometry could be assumed for other Ta cluster sizes too. The tendency of Ta clusters of this size to flatten and spread on the Pt surface is due to the stronger Pt–Ta interactions with respect to the cohesive energy of the Ta clusters.

In this regard, the differences in photoemission of Ta clusters of distinct size in Figure 2a,b could be interpreted as follows:

Considering 2D structures of Ta*_n_* clusters on Pt(111), their arrangement can be understood as a flat shell structure, where the central Ta atoms are more coordinated than the peripheral atoms, analogously to the trend observed from surface Ta atoms of reduced coordination on open surfaces. Here, an increase in BE is observed for Ta atoms of reduced coordination.^28,56^ The Ta–Ta residual coordination is proved by their calculated reciprocal distances being, in many cases, comparable to what observed for gas-phase Ta_8_ (Tables S3 and S4), with a general elongation of 0.02 nm to better fit with the underlying Pt(111) surface. In the following, str_1_(Ta_8_) is employed to further elucidate the XPS spectra in terms of distinct in-plane coordination number (CN) for Ta atoms in a Ta cluster. Based on the bond length found for cationic Ta clusters in the gas-phase (0.24–0.29 nm^57^)–we defined a truncation criterion of 0.35 nm for Pt(111) supported Ta clusters. Hence, Ta atoms displaying a Ta–Ta bond <0.35 nm, see Table S3, are considered as coordinated to the neighboring Ta atom. Consequently, a CN can be assigned to the individual Ta atoms (A1–A8) and they can be divided into four sets of in plane coordinated atoms: A1 (CN = 6), A7 (CN = 4), A8 (CN = 1) and the group of equivalent coordination A2–A6 (CN = 3), see Figure 2c.

In this sense, the contribution to the lowest BE (22.72 eV) arises from A1—the central tantalum atom of the cluster, see inset of Figure 2c. Vice versa, A8, which is adsorbed in a hollow site and solely coordinated to A7, displays a larger calculated BE (23.84 eV), a value comparable with what was reported for isolated Ta adatoms on Pt(111).^27^ All Ta atoms composing the hexagonal arrangement (A2–A7) around the central Ta atom exhibit intermediate BEs (23–23.4 eV), see Table S2. The dispersion of values in this range is most likely due to the effect of Ta–Ta interactions and different adsorption configurations with respect to the Pt(111) surface. Noteworthy, due to the additional coordination to A8, A7 displays the lowest BE of the hexagonal arrangement. Consequently, emissions from less coordinated Ta atoms are found to shift to higher BE and vice versa. Therefore, central atoms in a Ta_13_ cluster express a more bulk-like behavior than, for example, atoms in a Ta_4_ cluster, as they are neighbored by more Ta atoms and of higher coordination. Hence, the photoemission gradually broadens at the lower BE onset with increasing cluster size.^31,34^ Therefore, we can attribute the features in the Ta*_n_* spectra in Figure 2b to atoms with different CN in the clusters, whose density depends on the clusters size. Features at lower BE (less than 23.2 eV) can be attributed to atoms with higher CN (>4), which are more abundant for larger clusters, the main peak from the spectra in the range 23.2–23.6 eV is associated with atoms with intermediate CN (2–4) which are expected to be abundant in all the clusters due to their planar geometry, see the case of Ta_8_ and Ta_4_. Finally, the spectral region in the range 23.6–24.1 eV is attributed to atoms with the lowest CN (<2). This component is the highest in the case of Ta_1_, as expected from our interpretation, but is not zero for the larger clusters such as Ta_8_ where it is still possible to have atoms with low CN in the outer regions of the clusters, see Figure 2c. On the contrary, the small components in the region associated with intermediate and high CN in the Ta_1_ spectra are due to small agglomerates upon deposition of the adatoms.

Interestingly, the Bader charge expresses a similar trend as the BE with respect to the CN, see Figure 2c, and correlates with the Ta–Pt distance of the respective Ta atom, see Table S2. A1 is the most distant from the Pt surface and exhibits the lowest charge (+0.34|e|), while A8 is the closest to the Pt surface and possesses the highest charge (+1.17|e|). Consequently, an increase in both factors, the CN and Ta–Pt distance, contribute to decreasing the Bader charge and hence affect the oxidation state of the Ta cluster atoms. Furthermore, A8 presenting the highest charge while displaying the lowest CN is also in agreement with our findings concerning single Ta adatoms on Pt(111), where no possibility for in-plane coordination with Ta is given and an overall charge of +2 was identified.^27^

Among all of the Ta cluster sizes and the Ta adatoms, similar photoemission features are found on the high BE side (>24.1 eV) of the spectra. Considering the BE values of the Ta 4f_7/2_ photoemission of Ta-oxides in the literature (22.5–27 eV^6,16^), we attribute all the features observed above 24.1 eV to Ta-oxides–this region is indicated in Figure 2b in blue. The observed spectral structure in the range 24.1–25 eV suggests contributions from multiple oxidic structures or configurations, as inflection points can be observed. The distinct contributions to the Ta-oxide photoemission signals become more pronounced upon oxidation and have been discussed in ref (27). and in more detail in the following.

### Oxidation

**Figure 5:**
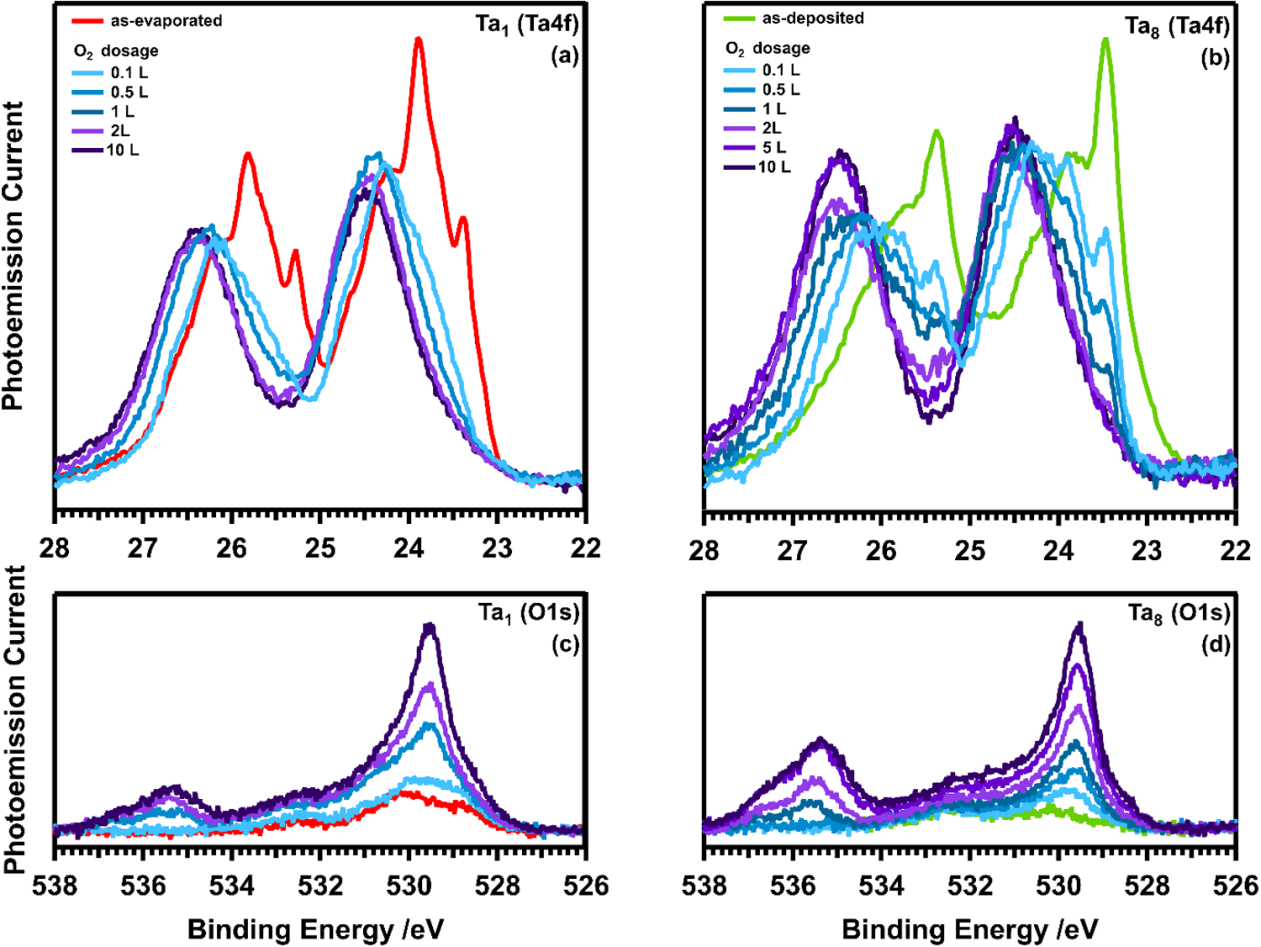
Sequence of XPS spectra showing the evolution of the Ta 4f (*h*υ = 150 eV) emissions, where the oxidation process sets in upon irradiation. (a) As-evaporated Ta atoms and (b) as-deposited Ta_8_ clusters on Pt(111) upon subsequent exposure to 0.1–10 L O_2_ at 40 K. The O 2s measurements for 0.1–10 L on Pt(111), see Figure S5, were subtracted from all oxidized Ta 4f spectra. The bottom spectra (c, d) display the corresponding evolution of the O 1s (*h*υ = 650 eV) region.

The different Ta cluster sizes and Ta adatoms were subsequently exposed to 0.1–10 L molecular O_2_ at 40 K (see Experimental Section). Upon oxidation emissions from both Ta 4f and O 2s orbitals can be identified in the region 22–28 eV. Although, the main features can be assigned to Ta 4f emissions, emissions from O 2s orbitals may also contribute to the measured spectra. This is especially true at higher O_2_ exposure (≥1 L), as evident from O 2s spectra recorded under the same conditions, in which the clean Pt(111) crystal was exposed to 0.1–10 L O_2_, see Figure S5. These O 2s spectra were subtracted from the Ta 4f spectra respective to the O_2_ dosage in order to account for the superposed emissions from chemisorbed molecular oxygen (O_chem_). The evolution of the respective Ta 4f and O 1s signals after deposition are displayed for Ta adatoms in Figure 5a,c and for Ta_8_ clusters in Figure 5b,d. Overall, in the Ta 4f emissions a gradual decline in emissions from both metallic Ta species of lower CN < 2 (BE 23.60–24.10 eV) and intermediate to higher CN > 2 (BE 23.60–22.00 eV), is observed, while features >24.10 eV originating from Ta-oxides become more pronounced. A similar progress is observed for all cluster sizes, as seen in Figure S4.

A low oxygen exposure of 0.1 L induces considerable changes in the Ta 4f and O 1s spectra of both Ta adatoms and Ta_8_ clusters. High CN features, i.e., components at BE < 23.2 eV in Figure 2c and associated with atoms with CN > 4, decrease considerably in intensity for Ta_8_ and even vanish in the case of Ta_1_, where they derived from agglomerates formed upon evaporation. Thus, an already low exposure to oxygen induces a rapid reaction, emphasizing the high oxygen affinity of Ta. The emissions related to atoms of low and intermediate CN (CN < 4 and BE = 23.2–24.1 eV) also decrease in intensity, but are sustained up to a higher exposure of 0.5 L in the Ta_1_ spectrum and 1 L in the case of the Ta_8_ spectrum. Thus, Ta_8_ seems to exhibit lower susceptibility to oxidation than Ta_1_.

It is noteworthy that, for the Ta_8_ clusters, the increase of photoemission signals of lower coordinated atoms could occur at the expense of higher coordinated ones. Thus, the sustained emission signal found at ∼23.6–23.8 eV for Ta_8_ could be reasoned by the formation of less coordinated Ta species upon cluster oxidation. In this scenario, an oxidative degradation of the Ta clusters is envisaged. Through abstraction—most likely initiated from Ta atoms of lower CN, i.e., from the periphery e.g., A8, atomic Ta species and concomitantly Ta cluster of reduced size would be yielded, resulting in Ta clusters comprising atoms of reduced coordination. Both Ta adatoms and Ta atoms of lower CN in a cluster can contribute to emissions found from 23.6 to 23.8 eV.

**Scheme 1:**
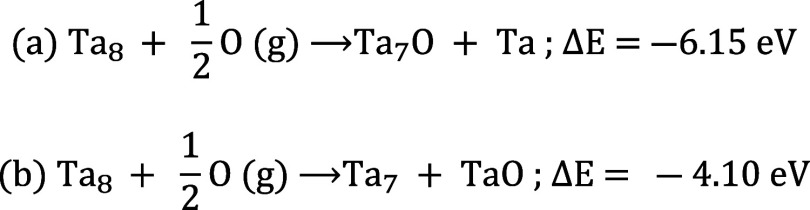
Oxidation Mechanism with (a) Ta Atom and (b) TaO Abstraction

This consideration agrees with the observations from the STM image in Figure 4b, where Ta fragments are observed after oxidation next to Ta-oxide islands, which are discussed later. Gas-phase oxidation studies on cationic Ta clusters support this picture: Upon oxidation, not solely intact Ta-oxo clusters form, but degradation takes place as well, leading to the abstraction of fragments from the cluster that is observed in the size range Ta_4_^+^–Ta_12_^+^.^58,59^ This oxidative cluster decomposition could account for the observed trend in emissions in parallel to the appearance of oxidized Ta species. In the gas-phase, for example, the oxidation of Ta_9_^+^ displays a considerably low activation energy (0.55 ± 0.03 kJ × mol^–1^) for the first degradation step.^59^ Considering that highly reactive atomic O is formed upon irradiation (Δ*H*_f_ (0 K) = 493.69 kJ/mol),^60^ and thus the oxidation process is activated, and given the small barriers for oxidation of Ta clusters that are obtained from gas-phase experiments, it can be concluded that the oxidation of Ta clusters and atoms (Δ*H*_f_ (297.15 K) = 192.46 kJ/mol)^61^ under our experimental conditions are kinetically achievable. In the following Scheme 1, two pathways, see also Figure S6, are proposed as tentative mechanisms for Ta cluster fragmentation upon reaction with atomic oxygen and have been treated computationally for the case of Ta_8_ clusters.

In the first mechanism, Scheme 1a, an oxygen atom is adsorbed on a Ta_8_ cluster, mimicking the initial stage of the oxidation, and this results in a partially oxidized Ta_7_O adduct and an isolated Ta atom bound to Pt(111). In the second mechanism, Scheme 1b, a TaO species forms and leaves a smaller, unoxidized Ta_7_ cluster. Simulation shows that both mechanisms are exothermic. However, the energetically more favorable mechanism is the abstraction of a Ta atom concomitant to oxidation of the remaining cluster of shrunk size as proposed in Scheme 1a. The resulting BE for Ta adatoms (23.62 and 23.77 eV for hcp and fcp hollow sites^27^) agrees with the photoemission signals observed in the oxidation spectra in Figure 5b. For the oxidized Ta_7_O cluster, the oxygen atom is three-coordinated to two peripheral and central Ta atoms. We observe that the peripheral Ta atoms directly bound to oxygen display a larger BE (23.80 eV) with respect to what is observed for unoxidized clusters. The central Ta atom also undergoes a substantial increase in BE (23.04 eV) compared to metallic Ta_8_. The mechanism suggests that the features observed in the Ta_8_ cluster spectra around 23.60 eV for higher O_2_ exposures up to 1 L could be attributed to (i) Ta adatoms, (ii) Ta_7_O clusters (Table S5) as well as to (iii) further degradation products of subsequently fragmented Ta-oxo-clusters displaying a reduced overall coordination.

The presence of metallic Ta atoms after oxidation can be explained experimentally, as the oxidation method does not supply oxygen continuously. As a result, atomic oxygen may be unavailable for reaction either because it is consumed during irradiation before Ta atoms can form due to fragmentation or because it is bound and too distant from the produced atoms (immobilized at 40 K). Our findings suggest the oxidation to proceed along mechanism (a), but with no complete fragmentation of the Ta clusters. More details are discussed in the Supporting Information (SI) in Figure S6.

Upon further exposure to oxygen (≥2 L), the Ta 4f spectra contain no longer metallic Ta contributions but solely the Ta-oxide-related ones at 24.50 eV. The corresponding O 1s (*h*υ = 650 eV) spectra, see Figures 5c,d and S5, indicate emissions from molecular O_chem_ on Pt(111) at 529.60–530.54 eV, atomic oxygen at 529.83 eV and physisorbed molecular O_2_ on Pt(111) at 534–538 eV.^62^ Ta-oxide-related photoemission signals around ∼530 eV are observed in the O 1s spectra of both, Ta adatoms and Ta_8_ clusters, directly after deposition and in line with the corresponding Ta 4f signatures (>24.1 eV). Compared to literature, BEs of ∼530 eV could be attributed to Ta_2_O_5_ (530.2–530.4 eV),^6,16^ which is also compatible with the high oxophilicity of Ta. With respect to Ta_7/2_ in the Ta 4f spectrum, an oxidation state of Ta^5+^, is expected to appear at BEs > 26 eV,^6,16^ which is clearly higher than the observed BEs (<25 eV). This might suggest that in our experiments the +5 state is not achieved, which would not be surprising based on several reported studies on the oxidation of metal clusters, which have shown that clusters are prone to oxidize in a 1:1 stoichiometry and hence to adopt a lower oxidation state compared to bulk materials.^29,31−33^ However, our present DFT simulations indicate that indeed the oxidation state of Ta is +5 and that the shift toward lower BEs is caused by the interaction with the Pt support.

### Ta-Oxide Islands

The observed XPS spectra of oxidized Ta adatoms and clusters at 40 K (see Figures S4 and S7) indicate the formation of a common oxidation product, independent of cluster size. To further elucidate, Ta_8_/Pt(111) samples were investigated by STM after oxidation and subsequent annealing, see Figure 4b,c. While the oxidation mechanism at low and high temperatures is not directly comparable, the XPS spectra after oxidation at 40 and 300 K display only small spectral differences, which are discussed later in this section; see Figure 7. In contrast to the stable and monodisperse clusters directly after deposition observed in Figure 4a, both measurements (b,c) indicate Ta agglomeration into oxidic islands. After oxidation, small Ta moieties are visible along the islands in Figure 4b. In contrast, these small moieties are no longer observed after subsequent annealing, and the number of islands decreases while they increase in size, Figure 4c. The islands do not have a particular shape and appear ragged. They consist of ∼50 atoms on average and are flat, similar to Ta clusters. The height profile reveals an apparent height of 0.07 nm with some second layer features of an apparent height of 0.13 nm. Hence, the Ta-oxide islands appear less high than the metallic Ta_8_ clusters (∼0.15 nm). It is intuitive to assign this island formation to the annealing process and cannot be excluded, however, as shown in the following, theoretical calculations suggest that for our samples island formations is also possible at 40 K. Experimentally, the formation of Ta-oxide islands at low temperatures, see Figure 7a, i.e., without thermal activation, can be reasoned as induced by the high oxophilicity of Ta. The nucleation into islands and lateral growth are known to often form the initial stage of oxidation, even at low temperatures.^63^ The Ta-oxide islands observed in STM likely represent a product similar to the common oxidation product observed for oxidized clusters at 40 K.

**Figure 6:**
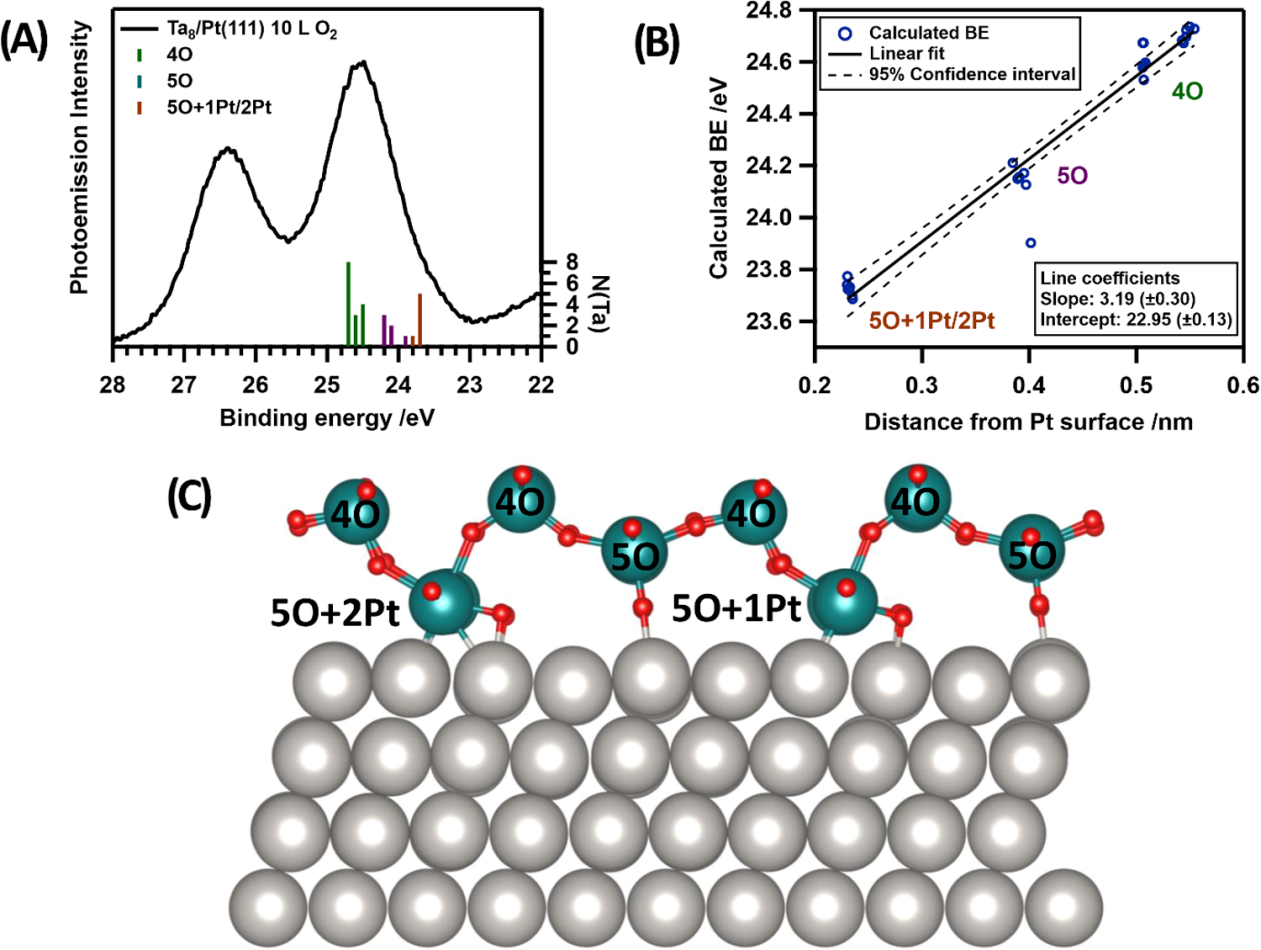
Computational model for Pt(111)-supported Ta_2_O_5_ islands with distinct surface and interface contributions (4O, 5O, 5O+1Pt, and 5O+2Pt) of Ta with O and Pt. (A) The computed BEs are compared to the experimental BEs and found in good agreement. The vertical axis on the right N(Ta) indicates the relative count of the single contributions to the film. (B) The BEs of all contributions versus their height with respect to the Pt(111) surface are reported. A pronounced perturbation from the Pt surface on the Ta BEs is observed that is particularly strong for Ta atoms in contact with Pt. (C) The simulated structure exhibits surface and interface contributions. The surface layer is constituted of four-coordinate 4O (24.5–24.9 eV) Ta species. The interface layer is in contact with the Pt surface and composed of three nonequivalent five-coordinated Ta species, whereas 5O (23.9–24.3 eV), 5O+1Pt and 5O+2Pt (23.6–23.8 eV) contributions can be distinguished.

We simulated oxidic Ta islands, adopting the Ta_2_O_5_ stoichiometry in the model. The resulting film represents the thinnest possible continuous model and comprises a two-dimensional layer of Ta_2_O_5_ building blocks, as shown in Figure 6. Upon coordination to Pt(111), the film exhibits a four-coordinate species (4O) at the surface and three nonequivalent five-coordinated species (5O, 5O+1Pt, 5O+2Pt) at the interface with Pt. The interface contributions differ in their local coordination regarding binding to the Pt(111) surface. Some of the Ta atoms bind to Pt through an O atom, while others adopt a direct coordination to the Pt surface. The corresponding side view in Figure 6C reveals 3 different types of Ta coordination: 4-fold coordination to oxygen atoms (4O), 5-fold coordination to oxygen atoms (5O), and 5-fold coordination to O envisaging also one (5O+1Pt) or two (5O+2Pt) bonds to the Pt(111) surface. The BEs of these types of Ta coordination are reported in Figure 6A, which are in good agreement with observed XPS spectra recorded at 40 K for oxidized samples. This supports the presence of agglomerates already at low temperatures without thermal activation. The graph in Figure 6B demonstrates that their calculated BEs correlate linearly with the Ta atoms’ height above to the Pt surface. This supports the strong perturbation exerted by the Pt surface on the Ta core levels, since the Ta atoms in contact to Pt display the smaller BEs (23.6–23.8 eV). A middle layer composed by 5O Ta species has intermediate BEs (23.9–24.3 eV). The top layer with four-coordinated Ta atoms shows the highest BEs (24.5–24.9 eV). The distance from Pt, thus, dominates over the influence of coordination and binding partners. This explains also the difficulty in associating a Ta oxidation state based on the observed Ta 4f BEs of the Ta-oxides.

**Figure 7:**
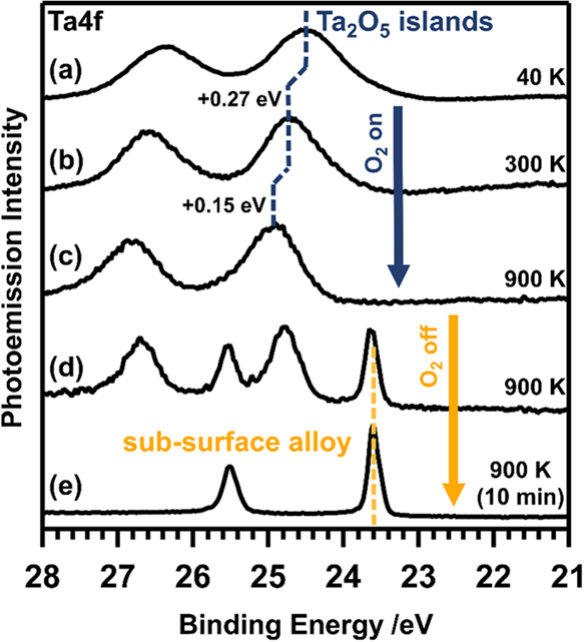
(a–c) Thermal evolution of the Ta 4f (*h*υ = 150 eV) emissions of the Ta-oxide islands from 40 to 900 K in an oxygen atmosphere (*p*(O_2_) = 1 × 10^–6^ mbar), followed by the conversion of the Ta-oxide islands into the Ta–Pt subsurface alloy under reductive UHV conditions at 900 K (c–e). Spectrum (d) represents a mixed-alloy state displaying emissions from both the Ta-oxide islands and the Ta–Pt subsurface alloy.

Annealing the Ta-oxide islands formed at low temperatures in an oxygen atmosphere (*p*_O_2__ = 1 × 10^–6^ mbar) to 300 K (+0.27 eV) and subsequently to 900 K (+0.15 eV) leads to a CLS and a narrowing of the Ta 4f photoemission signals, as shown in Figure 7a–c. The overall shift to higher BEs of +0.42 eV upon temperature increase points toward elongation of the Ta–O bonds with respect to the Ta_2_O_5_ phase at 40 K.^64^ The narrowing can be reasoned by converging to the minimum energy configuration, which becomes more accessible at higher temperatures. When the oxygen supply is turned off at 900 K, the photoemission signals from Ta-oxide islands gradually decrease, while that of the Ta–Pt subsurface alloy (23.59 eV) increases, as shown for the intermediate mixed-island-alloy state shown in Figure 7d. Finally, after <10 min of further annealing in UHV, the Ta 4f spectrum in Figure 7e displays solely emissions from the subsurface alloy. As demonstrated in our previous study, this alloy can be converted back into Ta-oxide islands in the presence of oxygen.^27^ Thus, two distinct chemical states of Ta at the Pt(111) surface can be interconverted by control of the environment (atmosphere and temperature), demonstrating the reversible duality of oxidative surface segregation and reductive subsurface intermixing.

## Conclusions

Size-selected Ta clusters soft-landed on a Pt(111) single crystal at 40 K were investigated using XPS and DFT. Concurrent photoemission signals from Ta clusters, adatoms and oxides have been spectroscopically observed upon deposition. The emissions of as-deposited Ta clusters display a size-specific trend. Emissions from Ta atoms with lower coordination, as in Ta_4_, shift to higher BE, while atoms of higher coordination, for example, central atoms in a Ta_13_ cluster, show more bulk-like behavior and shift to lower BE. This results in photoemission broadening at lower BE with increasing cluster size. The Bader charge displays a similar trend with BE and correlates with the Ta–Pt distance. Ta atoms far from the Pt surface display a low charge compared to a high charge for Ta atoms close to the surface. Both an increase in CN and Ta–Pt distance reduces the Bader charge of the Ta atom in the cluster, thereby influencing its oxidation state. At 40 K, all clusters are stable, and no beam damage is observed. In comparison, at 300 K, Ta_8_ is stable, as confirmed by STM, but Ta_4_ clusters show fragmentation induced by a thermodynamic drive for dispersion that the DFT calculations can rationalize. Notably, the stability of clusters larger than Ta_8_ can arguably be expected under these conditions. Furthermore, simulation and STM indicate an overall flat geometry of Ta_4_ and Ta_8_ clusters adsorbed to the Pt(111) surface, and based on the favorable interaction with Pt, this flat structure will hold for all Ta < 8 clusters at least. The origin of emissions concomitant to Ta adatoms in the cluster XPS spectra at 40 K is elucidated and reasoned to be related to the oxidation of Ta clusters. Slight traces of oxygen are sufficient to induce considerable changes to the XPS spectra that indicate the formation of Ta-oxides. The oxidation mechanism of the Ta_8_ clusters was treated computationally and appears to result via the formation of Ta-oxo-clusters with a concomitant abstraction of Ta atoms. A subsequent agglomeration of arguably mobile Ta-oxo-clusters of reduced size and Ta adatoms yields the final product of oxidation. STM combined with simulation reveals predominantly flat Ta-oxide islands, regardless of the cluster size, that form upon oxidation. At the low temperatures where it occurs, the process is not thermally but oxidatively driven. Spectroscopically, the Ta-oxide islands display a peculiar CLS inconsistent with values found in the literature for fully oxidized Ta^5+^. Complementary DFT calculations indicate distinct surface and interfacial Ta binding configurations in the Ta-oxide islands on Pt(111). Their BEs are strongly affected by the Pt-induced perturbation, which effectively reduces the observed BEs with respect to bulk Ta_2_O_5_, most prominently for Ta atoms in the vicinity of Pt. This outcome underlines the importance of fundamental insights into the interfacial interaction between Ta and Pt. The experimentally observed CLSs, together with the computational findings, emphasize that the measured Ta 4f BEs cannot be solely explained in terms of the Ta oxidation state. Last but not least, we demonstrate the environmentally driven duality between surface segregation and subsurface intermixing of Ta on the Pt support. We observe a strong tendency of Ta to segregate in the presence of oxygen to form Ta-oxide islands, while reductive annealing in UHV (>900 K) leads to intermixing of Ta into the Pt subsurface. Cyclic interconversion between the two states can be achieved and offers promising bimetallic material properties for potential application.

## Supporting Information Available

Simulated gas-phase cluster geometry (Figure S1), Ta_4_/Pt(111) STM image (Figure S2), first vs last Ta 4f XP scan all sizes (Figure S3), Ta 4f XP scans oxidation all sizes (Figure S4), O 1s/O 2s XP scans Pt(111) blanks (Figure S5), simulated oxidation mechanisms (Figure S6), Ta 4f XP scans (10 L) all sizes (Figure S7), O 1s/O 2s XP scan (10 L) references (Figure S8), Pt 4f XP scans Pt(111) blanks (Figure S9), computational values Ta_4_ (Table S1), computational values Ta_8_ (Table S2), Ta–Ta distances str_1_(Ta_8_)/Pt(111) (Table S3), Ta–Ta distances Ta_8_ gas-phase (Table S4), computational values Ta oxidation (Table S5), Ta–Ta distances str_2_(Ta_8_)/Pt(111) (Table S6) (PDF)

## Abbreviations

XPS: X-ray photoelectron spectroscopy
HR-XPS: high-resolution X-ray photoelectron spectroscopy
DFT: density functional theory
STM: scanning tunneling microscopy
UHV: ultrahigh vacuum
CLS: core-level shift
IER: internal experimental reference
BE: binding energy
*E*_ads_: adsorption energy
str: structure
CN: in-plane coordination number
O_chem_: chemisorbed molecular oxygen

## References

[ref1] Fleming R. M.; Lang D. V.; Jones C. D. W.; Steigerwald M. L.; Murphy D. W.; Alers G. B.; Wong Y.-H.; van Dover R. B.; Kwo J. R.; Sergent A. M. Defect dominated charge transport in amorphous Ta_2_O_5_ thin films. J. Appl. Phys. 2000, 88 (2), 850–862. 10.1063/1.373747.

[ref2] Novkovski N. Physical modeling of electrical and dielectric properties of high-k Ta_2_O_5_ based MOS capacitors on silicon. Facta Univ. - Ser.: Electron. Energ. 2014, 27 (2), 259–273. 10.2298/FUEE1402259N.

[ref3] Gao W.; Zhang Z.; Dou M.; Wang F. Highly Dispersed and Crystalline Ta_2_O_5_ Anchored Pt Electrocatalyst with Improved Activity and Durability Toward Oxygen Reduction: Promotion by Atomic-Scale Pt–Ta_2_O_5_ Interactions. ACS Catal. 2019, 9 (4), 3278–3288. 10.1021/acscatal.8b04505.

[ref4] Masud J.; Alam M. T.; Miah M. R.; Okajima T.; Ohsaka T. Enhanced electrooxidation of formic acid at Ta_2_O_5_-modified Pt electrode. Electrochem. Commun. 2011, 13 (1), 86–89. 10.1016/j.elecom.2010.11.020.

[ref5] Awaludin Z.; Sheng Moo J. G.; Okajima T.; Ohsaka T. TaO_x_-capped Pt nanoparticles as active and durable electrocatalysts for oxygen reduction. J. Mater. Chem. A 2013, 1 (46), 14754–14765. 10.1039/c3ta12492d.

[ref6] Kerrec O.; Devilliers D.; Groult H.; Marcus P. Study of dry and electrogenerated Ta_2_O_5_ and Ta/Ta_2_O_5_/Pt structures by XPS. Mater. Sci. Eng.: B 1998, 55 (1–2), 134–142. 10.1016/S0921-5107(98)00177-9.

[ref7] Yu J.; Chen G.; Li C. X.; Shafiei M.; Ou J.; Du Plessis J.; Kalantar-zadeh K.; Lai P. T.; Wlodarski W. Hydrogen gas sensing properties of Pt/Ta_2_O_5_ Schottky diodes based on Si and SiC substrates. Procedia Eng. 2010, 5, 147–151. 10.1016/j.proeng.2010.09.069.

[ref8] Skaja K.; Andrä M.; Rana V.; Waser R.; Dittmann R.; Baeumer C. Reduction of the forming voltage through tailored oxygen non-stoichiometry in tantalum oxide ReRAM devices. Sci. Rep. 2018, 8 (1), 10861 10.1038/s41598-018-28992-9. 30022129 PMC6052165

[ref9] Yoo H. K.; Lee S. B.; Lee J. S.; Chang S. H.; Yoon M. J.; Kim Y. S.; Kang B. S.; Lee M.-J.; Kim C. J.; Kahng B.; Noh T. W. Conversion from unipolar to bipolar resistance switching by inserting Ta_2_O_5_ layer in Pt/TaOx/Pt cells. Appl. Phys. Lett. 2011, 98 (18), 183507 10.1063/1.3587809.

[ref10] Sakamoto T.; Lister K.; Banno N.; Hasegawa T.; Terabe K.; Aono M. Electronic transport in Ta_2_O_5_ resistive switch. Appl. Phys. Lett. 2007, 91 (9), 092110 10.1063/1.2777170.

[ref11] Lin J.; Masaaki N.; Tsukune A.; Yamada M. Ta_2_O_5_ thin films with exceptionally high dielectric constant. Appl. Phys. Lett. 1999, 74 (16), 2370–2372. 10.1063/1.123854.

[ref12] Wang K.; Jia Y. Model and simulation of bilayered tantalum-oxide (Pt/Ta_2_O_5_/TaO_x_/Pt) memristor. J. Phys.: Conf. Ser. 2022, 2295 (1), 12005 10.1088/1742-6596/2295/1/012005.

[ref13] Parshina L.; Novodvorsky O.; Khramova O.; Gusev D.; Polyakov A.; Cherebilo E. Tuning the resistive switching in tantalum oxide-based memristors by oxygen pressure during low temperature laser synthesis. Chaos, Solitons Fractals 2022, 161, 112384 10.1016/j.chaos.2022.112384.

[ref14] Parshina L.; Novodvorsky O.; Khramova O.; Gusev D.; Polyakov A.; Mikhalevsky V.; Cherebilo E. Laser synthesis of non-volatile memristor structures based on tantalum oxide thin films. Chaos, Solitons Fractals 2021, 142, 110460 10.1016/j.chaos.2020.110460.

[ref15] Sharath S. U.; Joseph M. J.; Vogel S.; Hildebrandt E.; Komissinskiy P.; Kurian J.; Schroeder T.; Alff L. Impact of oxygen stoichiometry on electroforming and multiple switching modes in TiN/TaO_x_/Pt based ReRAM. Appl. Phys. Lett. 2016, 109 (17), 173503 10.1063/1.4965872.

[ref16] Simpson R.; White R. G.; Watts J. F.; Baker M. A. XPS investigation of monatomic and cluster argon ion sputtering of tantalum pentoxide. Appl. Surf. Sci. 2017, 405, 79–87. 10.1016/j.apsusc.2017.02.006.

[ref17] Benito N.; Palacio C. Nanostructuring of Ta_2_O_5_ surfaces by low energy Ar^+^ bombardment. Appl. Surf. Sci. 2015, 351, 753–759. 10.1016/j.apsusc.2015.05.143.

[ref18] Atanassova E.; Spassov D. X-ray photoelectron spectroscopy of thermal thin Ta_2_O_5_ films on Si. Appl. Surf. Sci. 1998, 135 (1–4), 71–82. 10.1016/S0169-4332(98)00278-5.

[ref19] Zier M.; Oswald S.; Reiche R.; Wetzig K. XPS investigations of thin tantalum films on a silicon surface. Anal. Bioanal. Chem. 2003, 375 (7), 902–905. 10.1007/s00216-003-1788-2. 12707758

[ref20] Li Y.; Sanna S.; Norrman K.; Christensen D. V.; Pedersen C. S.; Lastra J. M. G.; Traulsen M. L.; Esposito V.; Pryds N. Tuning the stoichiometry and electrical properties of tantalum oxide thin films: 38% LG ratio. Appl. Surf. Sci. 2019, 470, 1071–1074. 10.1016/j.apsusc.2018.11.153.

[ref21] Medicherla V. R. R.; Majumder S.; Paramanik D.; Varma S. Formation of self-organized Ta nano-structures by argon ion sputtering of Ta foil: XPS and AFM study. J. Electron Spectrosc. Relat. Phenom. 2010, 180 (1–3), 1–5. 10.1016/j.elspec.2010.02.006.

[ref22] Perez I.; Sosa V.; Perera F. G.; Galindo J. T. E.; Enríquez-Carrejo J. L.; González P. G. M. Effect of ion bombardment on the chemical properties of crystalline tantalum pentoxide films. Vacuum 2019, 165, 274–282. 10.1016/j.vacuum.2019.04.037.

[ref23] Bradley R. M.; Harper J. M. E. Theory of ripple topography induced by ion bombardment. J. Vac. Sci. Technol., A 1988, 6 (4), 2390–2395. 10.1116/1.575561.

[ref24] Bradley R. M.; Shipman P. D. Spontaneous pattern formation induced by ion bombardment of binary compounds. Phys. Rev. Lett. 2010, 105 (14), 145501 10.1103/PhysRevLett.105.145501. 21230842

[ref25] Lewin E.; Counsell J.; Patscheider J. Spectral artefacts post sputter-etching and how to cope with them – A case study of XPS on nitride-based coatings using monoatomic and cluster ion beams. Appl. Surf. Sci. 2018, 442, 487–500. 10.1016/j.apsusc.2018.02.191.

[ref26] Mathieu H. J.; Landolt D. On the influence of crater geometry on depth resolution of AES and XPS profiles of tantalum oxide films. Surf. Interface Anal. 1983, 5 (2), 77–82. 10.1002/sia.740050205.

[ref27] Bertrang K.; Hinke T.; Kaiser S.; Knechtges M.; Loi F.; Sbuelz L.; Lacovig P.; Bignardi L.; Esch F.; Baraldi A.; et al. Unraveling the interaction of Ta atoms with Pt(111). Surf. Interfaces 2025, 56, 105640 10.1016/j.surfin.2024.105640.

[ref28] Riffe D. M.; Wertheim G. K. Ta(110) surface and subsurface core-level shifts and 4f_7/2_ line shapes. Phys. Rev. B 1993, 47 (11), 6672 10.1103/PhysRevB.47.6672. 10004638

[ref29] Loi F.; Pozzo M.; Sbuelz L.; Bignardi L.; Lacovig P.; Tosi E.; Lizzit S.; Kartouzian A.; Heiz U.; Alfè D.; Baraldi A. Oxidation at the sub-nanoscale: oxygen adsorption on graphene-supported size-selected Ag clusters. J. Mater. Chem. A 2022, 10 (27), 14594–14603. 10.1039/D2TA02539F.

[ref30] Argyle M.; Bartholomew C. Heterogeneous Catalyst Deactivation and Regeneration: A Review. Catalysts 2015, 5 (1), 145–269. 10.3390/catal5010145.

[ref31] Loi F.; Bignardi L.; Perco D.; Berti A.; Lacovig P.; Lizzit S.; Kartouzian A.; Heiz U.; Alfè D.; Baraldi A. Unveiling Inequality of Atoms in Ultrasmall Pt Clusters: Oxygen Adsorption Limited to the Uppermost Atomic Layer. Small Struct. 2024, 5 (11), 2400250 10.1002/sstr.202400250.

[ref32] Loi F.; Pozzo M.; Sbuelz L.; Bignardi L.; Lacovig P.; Tosi E.; Lizzit S.; Kartouzian A.; Heiz U.; Larciprete R.; et al. Breakdown of the correlation between oxidation states and core electron binding energies at the sub-nanoscale. Appl. Surf. Sci. 2023, 619, 156755 10.1016/j.apsusc.2023.156755.

[ref33] Perco D.; Loi F.; Bignardi L.; Sbuelz L.; Lacovig P.; Tosi E.; Lizzit S.; Kartouzian A.; Heiz U.; Baraldi A. The highest oxidation state observed in graphene-supported sub-nanometer iron oxide clusters. Commun. Chem. 2023, 6 (1), 61 10.1038/s42004-023-00865-x. 37012362 PMC10070315

[ref34] Sbuelz L.; Loi F.; Pozzo M.; Bignardi L.; Nicolini E.; Lacovig P.; Tosi E.; Lizzit S.; Kartouzian A.; Heiz U.; et al. Atomic Undercoordination in Ag Islands on Ru(0001) Grown via Size-Selected Cluster Deposition: An Experimental and Theoretical High-Resolution Core-Level Photoemission Study. J. Phys. Chem. C 2021, 125 (17), 9556–9563. 10.1021/acs.jpcc.1c02327. PMC8279646 34276855

[ref35] Heiz U.; Vanolli F.; Trento L.; Schneider W.-D. Chemical reactivity of size-selected supported clusters: An experimental setup. Rev. Sci. Instrum. 1997, 68 (5), 1986–1994. 10.1063/1.1148113.

[ref36] Goldoni A.; Baraldi A.; Comelli G.; Esch F.; Larciprete R.; Lizzit S.; Paolucci G. Morphology and magnetic properties of thin films of Rh on highly oriented pyrolitic graphite. Phys. Rev. B 2000, 63 (3), 035405 10.1103/PhysRevB.63.035405.

[ref37] Bianchettin L.; Baraldi A.; de Gironcoli S.; Vesselli E.; Lizzit S.; Petaccia L.; Comelli G.; Rosei R. Core level shifts of undercoordinated Pt atoms. J. Chem. Phys. 2008, 128 (11), 114706 10.1063/1.2841468. 18361600

[ref38] Nečas D.; Klapetek P. Gwyddion: an open-source software for SPM data analysis. Open Phys. 2012, 10 (1), 181–188. 10.2478/s11534-011-0096-2.

[ref39] Kresse G.; Hafner J. Ab initio molecular dynamics for liquid metals. Phys. Rev. B 1993, 47 (1), 558 10.1103/PhysRevB.47.558. 10004490

[ref40] Kresse G.; Furthmüller J. Efficient iterative schemes for ab initio total-energy calculations using a plane-wave basis set. Phys. Rev. B 1996, 54 (16), 11169 10.1103/PhysRevB.54.11169. 9984901

[ref41] Kresse G.; Joubert D. From ultrasoft pseudopotentials to the projector augmented-wave method. Phys. Rev. B 1999, 59 (3), 1758 10.1103/PhysRevB.59.1758.

[ref42] Blöchl P. E. Projector augmented-wave method. Phys. Rev. B 1994, 50 (24), 17953 10.1103/PhysRevB.50.17953. 9976227

[ref43] Perdew J. P.; Burke K.; Ernzerhof M. Generalized Gradient Approximation Made Simple. Phys. Rev. Lett. 1996, 77 (18), 3865 10.1103/PhysRevLett.77.3865. 10062328

[ref44] Weinert M.; Watson R. E. Core-level shifts in bulk alloys and surface adlayers. Phys. Rev. B 1995, 51 (23), 17168 10.1103/PhysRevB.51.17168. 9978730

[ref45] Grimme S.; Antony J.; Ehrlich S.; Krieg H. A consistent and accurate ab initio parametrization of density functional dispersion correction (DFT-D) for the 94 elements H-Pu. J. Chem. Phys. 2010, 132 (15), 154104 10.1063/1.3382344. 20423165

[ref46] Grimme S.; Ehrlich S.; Goerigk L. Effect of the damping function in dispersion corrected density functional theory. J. Comput. Chem. 2011, 32 (7), 1456–1465. 10.1002/jcc.21759. 21370243

[ref47] Xiao W.; Huang X.; Song W.; Yang Y.; Herng T. S.; Xue J. M.; Feng Y. P.; Ding J. High catalytic activity of oxygen-induced (200) surface of Ta_2_O_5_ nanolayer towards durable oxygen evolution reaction. Nano Energy 2016, 25, 60–67. 10.1016/j.nanoen.2016.04.020.

[ref48] Guillot C.; Chauveau D.; Roubin P.; Lecante J.; Desjonquères M. C.; Tréglia G.; Spanjaard D. Core level spectroscopy of the low index faces of Tantalum. Surf. Sci. 1985, 162 (1–3), 46–50. 10.1016/0039-6028(85)90874-X.

[ref49] van der Veen J. F.; Himpsel F. J.; Eastman D. E. Chemisorption-induced 4f -core-electron binding-energy shifts for surface atoms of W(111), W(100), and Ta(111). Phys. Rev. B 1982, 25 (12), 7388 10.1103/PhysRevB.25.7388.

[ref50] Li X.; Chen Y.; Basnet P.; Luo J.; Wang H. Probing the properties of size dependence and correlation for tantalum clusters: geometry, stability, vibrational spectra, magnetism, and electronic structure. RSC Adv. 2019, 9 (2), 1015–1028. 10.1039/C8RA09240K. 35517637 PMC9059546

[ref51] Ruban A. V.; Skriver H. L.; Nørskov J. K. Surface segregation energies in transition-metal alloys. Phys. Rev. B 1999, 59 (24), 15990 10.1103/PhysRevB.59.15990.

[ref52] Krupski K.; Moors M.; Jóźwik P.; Kobiela T.; Krupski A. Structure Determination of Au on Pt(111) Surface: LEED, STM and DFT Study. Materials 2015, 8 (6), 2935–2952. 10.3390/ma8062935.

[ref53] Wertheim G. K. Core-electron binding energies in free and supported metal clusters. Z. Phys. B: Condens. Matter 1987, 66 (1), 53–63. 10.1007/BF01312762. 9944577

[ref54] Schaub R.; Jödicke H.; Brunet F.; Monot R.; Buttet J.; Harbich W. Decorated Ag_19_ on Pt(111) or the ″rare gas necklace″. Phys. Rev. Lett. 2001, 86 (16), 3590 10.1103/PhysRevLett.86.3590. 11328030

[ref55] Fukamori Y.; König M.; Yoon B.; Wang B.; Esch F.; Heiz U.; Landman U. Fundamental Insight into the Substrate-Dependent Ripening of Monodisperse Clusters. ChemCatChem 2013, 5 (11), 3330–3341. 10.1002/cctc.201300250.

[ref56] Riffe D. M.; Hale W.; Kim B.; Erskine J. L. Conduction-electron screening in the bulk and at low-index surfaces of Ta metal. Phys. Rev. B 1995, 51 (16), 11012 10.1103/PhysRevB.51.11012. 9977804

[ref57] Fielicke A.; Gruene P.; Haertelt M.; Harding D. J.; Meijer G. Infrared spectroscopy and binding geometries of oxygen atoms bound to cationic tantalum clusters. J. Phys. Chem. A 2010, 114 (36), 9755–9761. 10.1021/jp102084n. 20504043

[ref58] Eckhard J. F.; Neuwirth D.; Panosetti C.; Oberhofer H.; Reuter K.; Tschurl M.; Heiz U. Consecutive reactions of small, free tantalum clusters with dioxygen controlled by relaxation dynamics. Phys. Chem. Chem. Phys. 2017, 19 (8), 5985–5993. 10.1039/C6CP07631A. 28181608

[ref59] Eckhard J. F.; Neuwirth D.; Tschurl M.; Heiz U. From oxidative degradation to direct oxidation: size regimes in the consecutive reaction of cationic tantalum clusters with dioxygen. Phys. Chem. Chem. Phys. 2017, 19 (17), 10863–10869. 10.1039/C7CP01293D. 28401239

[ref60] Ruscic B.; Feller D.; Peterson K. A. Active Thermochemical Tables: dissociation energies of several homonuclear first-row diatomics and related thermochemical values. Theor. Chem. Acc. 2014, 133, 1415 10.1007/s00214-013-1415-z.

[ref61] National Institute of Standards and Technology Journal of Physical and Chemical Reference Data Monographs, American Institute of Physics, 1998.

[ref62] Puglia C.; Nilsson A.; Hernnäs B.; Karis O.; Bennich P.; Mårtensson N. Physisorbed, chemisorbed and dissociated O_2_ on Pt(111) studied by different core level spectroscopy methods. Surf. Sci. 1995, 342 (1–3), 119–133. 10.1016/0039-6028(95)00798-9.

[ref63] Franchy R. Growth of thin, crystalline oxide, nitride and oxynitride films on metal and metal alloy surfaces. Surf. Sci. Rep. 2000, 38 (6–8), 195–294. 10.1016/S0167-5729(99)00013-8.

[ref64] Pedersen C. S.; Chang J. H.; Li Y.; Pryds N.; Garcia Lastra J. M. Phase separation in amorphous tantalum oxide from first principles. APL Mater. 2020, 8 (7), 071108 10.1063/5.0011390.

