# Supplementary material for: The Interaction of Sub-Monolayer Ta Adatoms and Clusters with Oxygen at the Pt(111) Interface
Source: J Phys Chem C Nanomater Interfaces. 2025 Mar 21;129(13):6511–23. doi: 10.1021/acs.jpcc.5c00699 (PMC11973982; doi:10.1021/acs.jpcc.5c00699)
Supplement: Supplementary file 1 — jp5c00699_si_001.pdf [file jp5c00699_si_001.pdf]

Supporting Information

The Interaction of sub-Monolayer Ta Adatoms and Clusters with Oxygen at the Pt(111) Interface

Kevin Bertrang‡[a], Tobias Hinke‡[a], Sebastian Kaiser[a], Matthias Knechtges[a], Federico Loi[b], Paolo Lacovig[c], Mirali Jahangirzadeh Varjovi[d], Friedrich Esch[a], Alessandro Baraldi[bc], Sergio Tosoni[d], Aras Kartouzian[a], Ueli Heiz[a]*

[a] TUM School of Natural Sciences, Department of Chemistry, Chair of Physical Chemistry, Technical University of Munich; Catalysis Research Center, Technical University of Munich, Garching D-85748, Germany; [b]Department of Physics, University of Trieste, 34127 Trieste, Italy; [c]Elettra-Sincrotrone Trieste, 34149 Trieste, Italy; [d]Dipartimento di Scienza dei Materiali, Università di Milano Bicocca, 20125 Milano, Italy

Ta_4 and Ta_8 cluster gas-phase structures

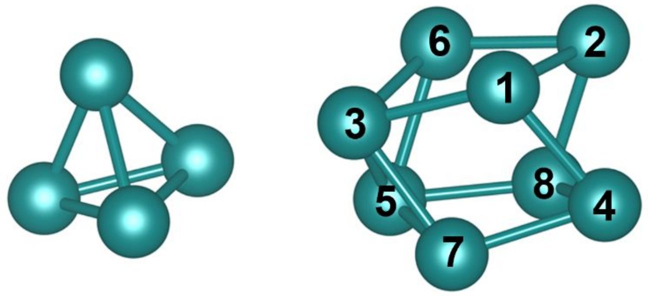

Figure S1. Calculated structures of gas-phase Ta_4 (left) and Ta_8 (right) clusters. The numbering of the atoms of Ta_8 relate to the bond distances reported in Table S4, see (A_g1-8), where the index g refers to an atom from a gas-phase structure.

STM image of the Ta_4 clusters on Pt(111) at 300 K

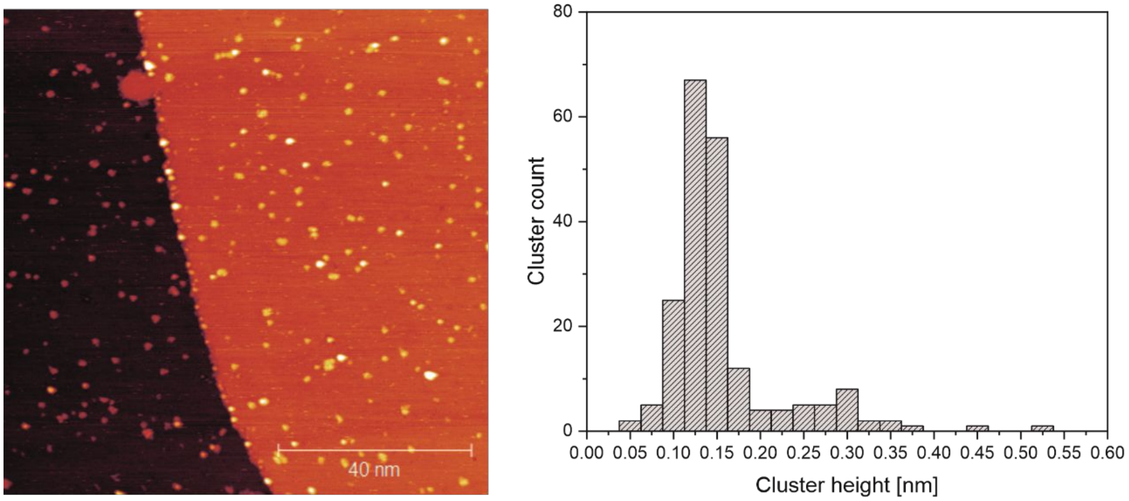

Figure S2. Left side: Ta_4 clusters deposited on Pt(111) at 300 K with a cluster coverage of 0.13% clusters/ML_{Pt}. The Ta clusters seem partially to sinter upon deposition and are thus not fully monodisperse. Additionally, some tend to accumulate at the Pt step edges. Besides intact Ta_4 and sintered Ta clusters, small fragments of unspecific size can be observed. Right side: Evaluation of the imaged cluster heights yields a bimodal distribution. While Ta_4 appears with an average height of 0.12 nm, an additional feature at roughly twice the height (~0.3 nm) indicates sintered particles.

Ta $4f_{7/2}$ spectra of all cluster sizes as-deposited on Pt(111) at 40 K:

First vs last XP scan

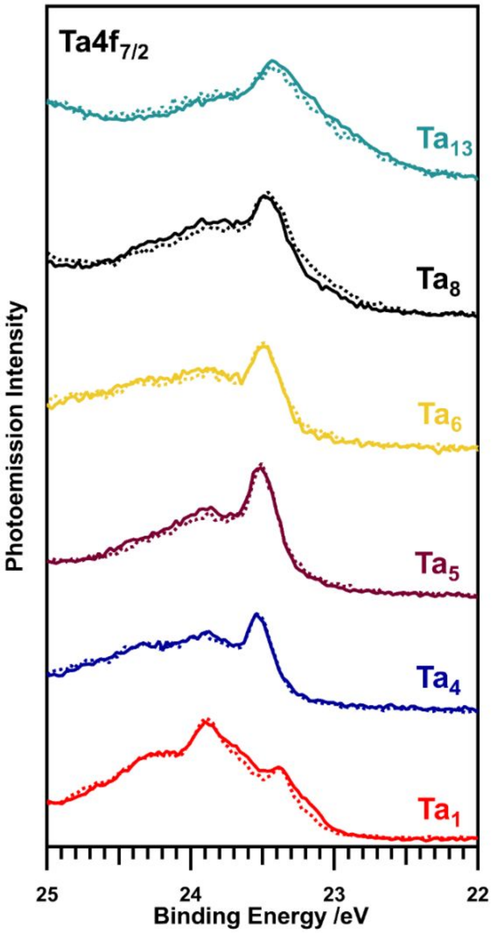

Figure S3. Stacked overview of the Ta 4f XP spectra (hυ=150 eV) of Ta adatoms after evaporation and Ta_n clusters (n=4, 5, 6, 8, 13) after deposition on Pt(111) at 40 K. For each cluster size (Ta atoms (red), Ta_4 (dark blue), Ta_5 (brown), Ta_6 (yellow), Ta_8 (black) and Ta_{13} (turquoise)), a series of five consecutive measurements was conducted to evaluate the changes induced by the incident X-ray beam. The first and the last spectrum are displayed (solid, and dotted lines, respectively).

No considerable beam damage was detected and hence, all clusters are assumed stable upon irradiation.

Oxidation of the different Ta cluster sizes on Pt(111) at 40 K

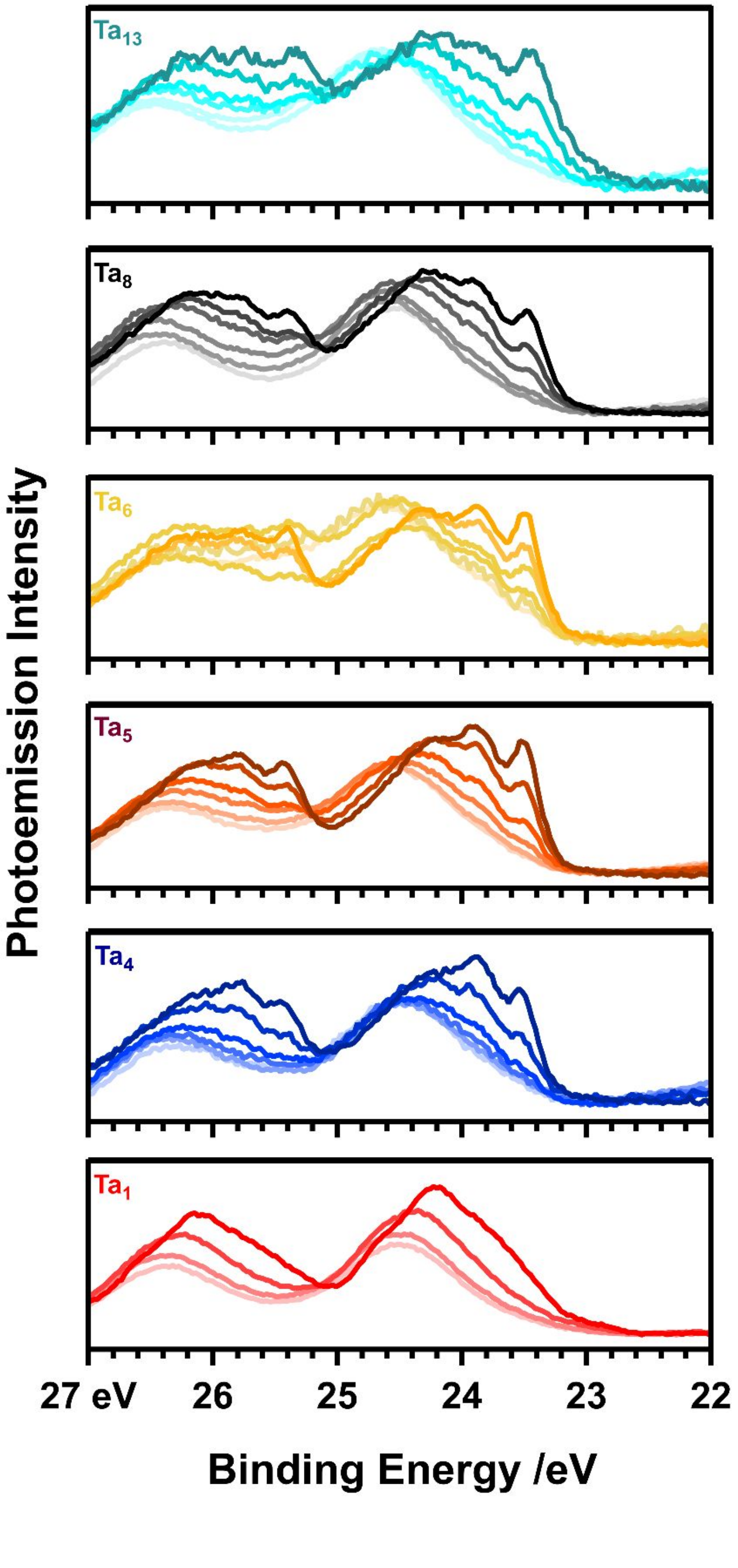

Figure S4. Stacked overview of the Ta 4f XP spectra (hυ=150 eV) of evaporated Ta atoms and deposited Ta_n clusters (n=4, 5, 6, 8, 13) on Pt(111) at 40 K as they evolve in the presence of O_2 (0.1-10 L). For each cluster size (Ta adatoms (red), Ta_4 (dark blue), Ta_5 (brown), Ta_6 (yellow), Ta_8 (black) and Ta_{13} (turquoise)), an exposure of only 0.1 L O_2 (indicated by the color gradients) induces already considerable changes in the XP spectra. After an exposure of 5 L O_2, the oxidation is completed, yielding similar spectra for all cluster sizes.

O 2s photoemission signals overlap with the Ta 4f signals. These contributions of distinct oxygen phases on Pt(111) are shown in Figures S5 and S8. O 2s emissions from atomic oxygen (21 eV), physisorbed molecular O_2 (21.3 eV and 31 eV), and chemisorbed molecular O_2 (O_{chem}) on Pt(111) (25 eV) can be identified[1]. Especially the O_{chem} photoemission signal is superposed with Ta 4f emissions, and its accumulation can account for the changes in the Ta 4f region at higher O_2 exposure. Additionally, photoemission from H_2O, present in the residual gas background and adsorbed on Pt(111), is observed in minor traces in the O 1s spectra (~532 eV). Its corresponding O 2s signal at 25.7-26.1 eV[2] can further contribute to the background of the Ta 4f region. The effect of O_2 exposure on the P4 4f spectra is displayed in Figure S9.

Evolution of the O 1s and O 2s XP spectra upon exposure of Pt(111) to O_2

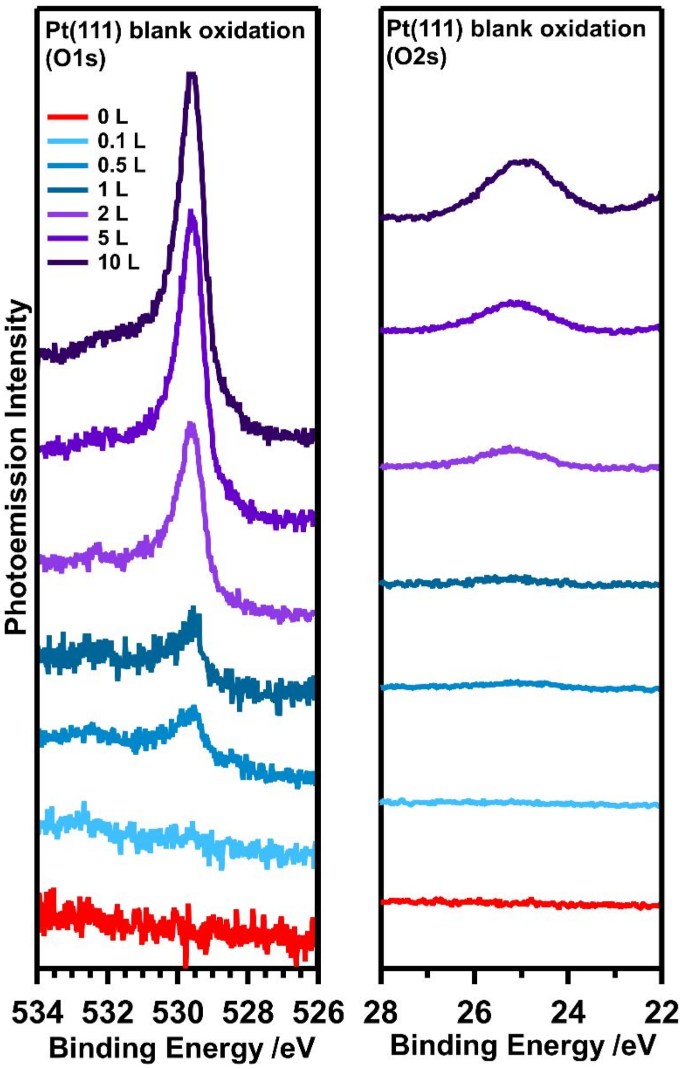

Figure S5. Left side: Evolution of the O 1s XP spectra (hυ=650 eV) when dosing 0.1-10 L O_2 on Pt(111) at 40 K. Right side: Corresponding O 2s XP spectra (hυ=150 eV). The gradual increase of the O_{chem} and H_2O signals can be followed (for the assignment see Fig. S8).

Simulated oxidation mechanism and structures

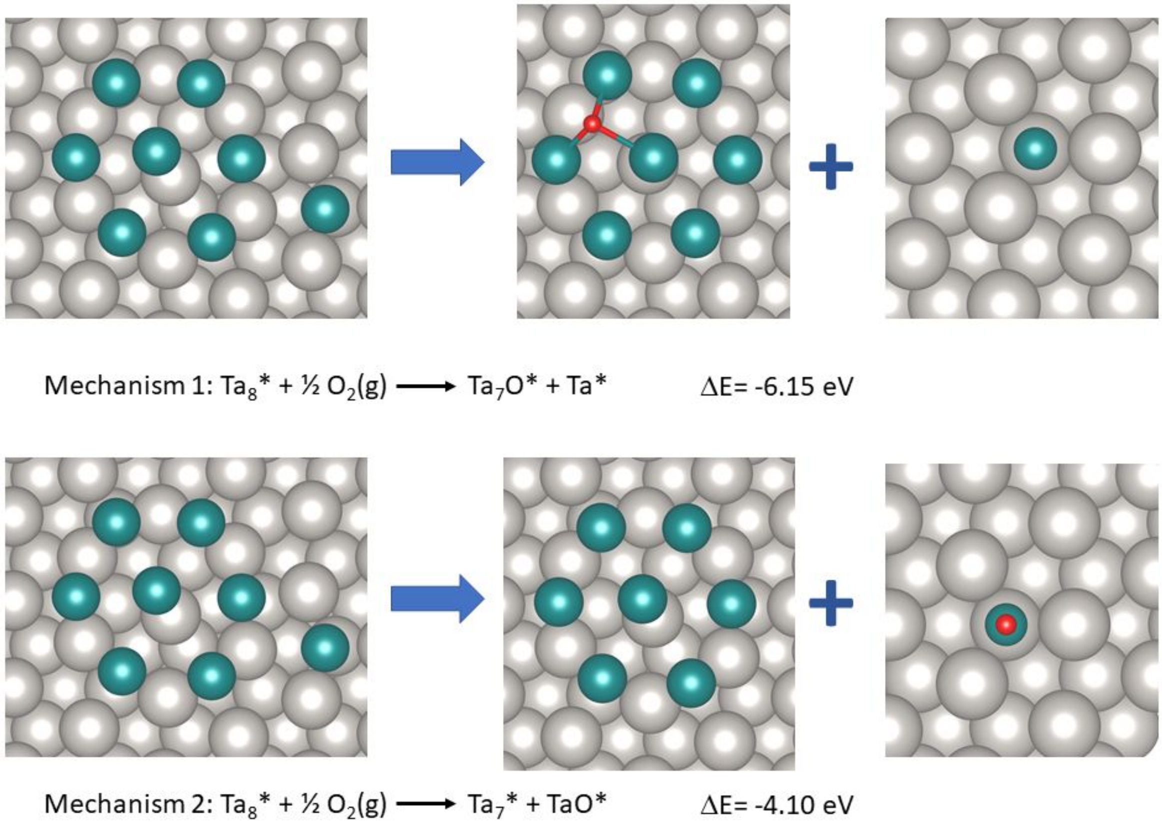

Figure S6. Schematic representation of the possible oxidation mechanisms for Ta_8 clusters (see Scheme 1 in the main text).

Both mechanisms could imply the presence of TaO fragments, either as a direct product or as a result of further fragmentation. Computationally, the adsorption of a variable oxygen loading to a Ta adatom in a hcp site on the Pt(111) surface leads to stable TaO_x complexes (x=1-2). However, as seen in Table S5, those species' calculated Ta 4f BEs all remarkably undergo a CLS towards lower BE (22.29-22.85 eV) compared to metallic Ta adatoms on Pt(111). Experimentally, such species are not identified, hinting at the absence of small TaO_x complexes. Thus, it is proposed that the oxidation follows mechanism (a), though not resulting in the complete fragmentation of

the Ta clusters. The Ta-oxides-related emissions found at BEs of >24 eV are observed concomitantly to cluster fragmentation. Interestingly, the computations indicate that the formation of new Ta-O bonds does not only increase the oxidation state of the Ta clusters (with respect to the Pt-induced oxidation) but also pushes the respective Ta atoms away from the Pt surface. This elongation of the Ta-Pt bonds upon oxidation (see R(Ta-Pt) in Table S5) weakens the metallic Ta-Pt interaction. Since TaO_x (x=1-2) species are not observed upon oxidation of Ta_8, but would be expected for complete fragmentation, the formation of more extended Ta-oxides (at BEs of >24 eV) can be understood as an agglomeration of oxidic Ta species (≤Ta_7O and >TaO_{1-2}), which diffuse on the surface due to the exothermicity of the oxidation reaction and reduced interaction with the support due to bond elongation. Nevertheless, the intermediate formation of short-lived TaO_x (x=1-2) species cannot be ruled out.

Oxidation of the different Ta cluster sizes on Pt(111) at 40 K with 10 L O_2

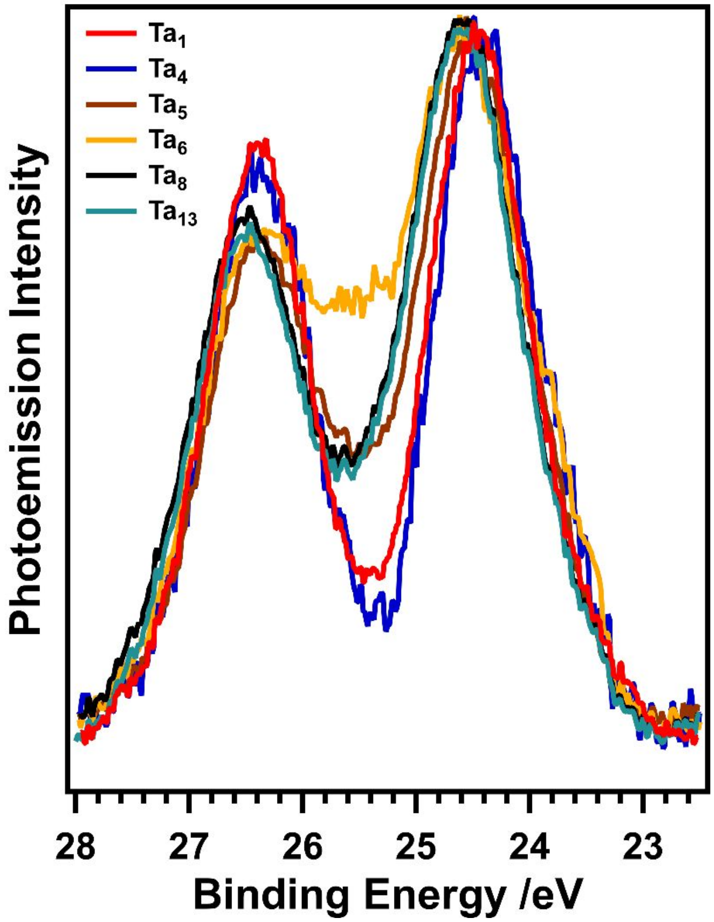

Figure S7. Ta 4f spectra (hυ=150 eV) of $Ta_{1\text{-}13}$ after exposure to 10 L O_2 after subtraction of the respective O2 s blank measurement (see Figure S5) to account for O_{chem} related contributions in the region of the Ta 4f emissions.

Upon oxidation of the $Ta_{1\text{-}13}$ clusters a gradual evolution of the XPS spectra is observed, see Figure S4. The final product of oxidation (10 L) for each cluster size is displayed here in Figure S7. The corresponding O 2s blank measurement for 10 L (see Figure S5) was subtracted to account for O_{chem} related emissions. Comparable Ta $4f_{7/2}$ line shapes (broad Gaussian) displaying emissions in the range 23-25.5 eV are observed for all sizes. The differences between

the spectra are mainly observed for emissions found around 25.5 eV, as e.g., most pronounced for Ta_6. The absence of a respective spin orbit component strictly excludes any Ta species as the origin. These features might arise from the accumulation of H_2O and OH (25.7-26.1 eV) on the surface at low temperatures and may vary for the different cluster sizes as a function of deposition and measurement time and the relative background pressure in the cluster source and UHV chamber. The underling O 2s contribution also affects the spin orbit ratio (4:3), as it does not contribute equally to the multiplet. Hence, it can lead to a slight shift to higher BE compared to the sizes which display no such features in the oxidation product, for example Ta_4.

O 1s and O 2s photoemission spectra upon exposure of Pt(111) to 10 L O_2

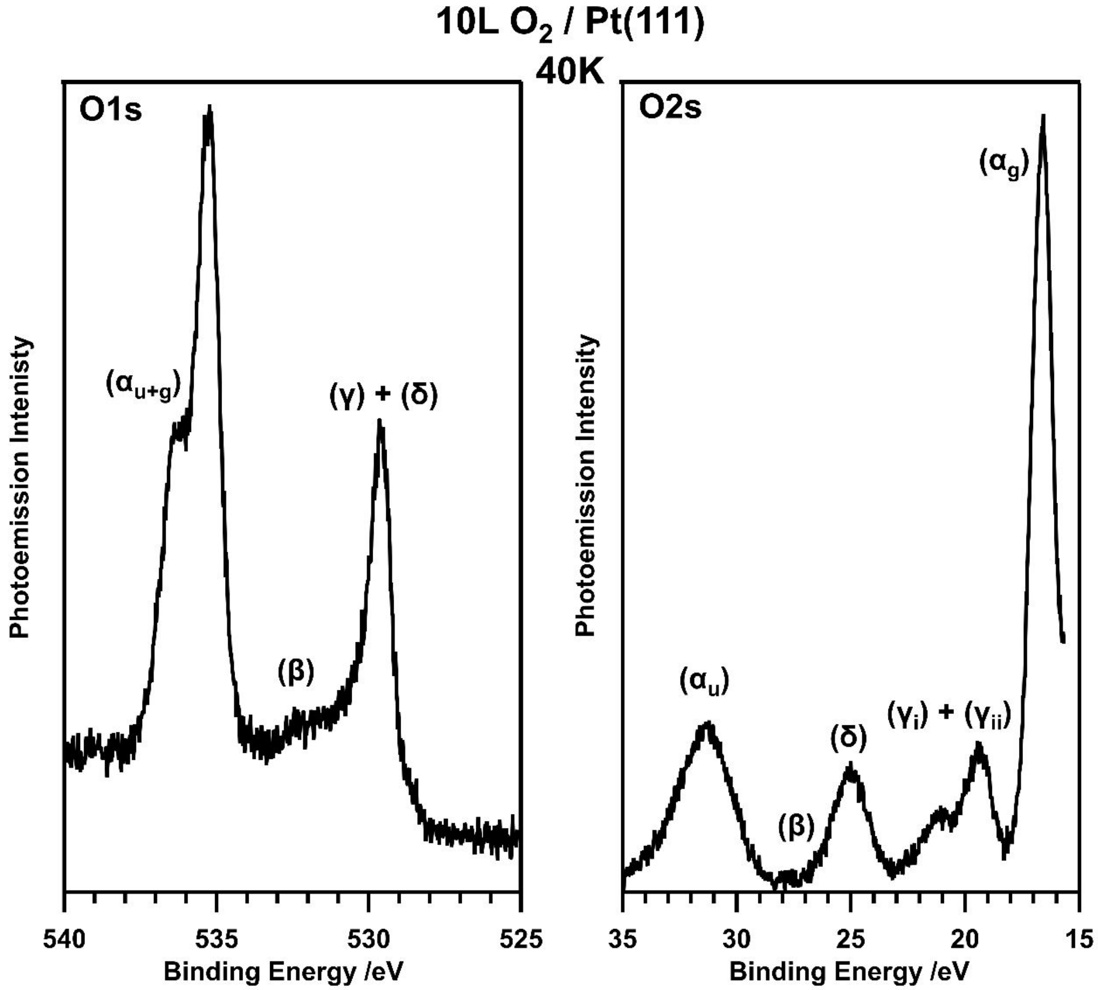

Figure S8. Left side: O 1s XP spectrum (hυ=650 eV) after dosing 10 L O_2 on Pt(111) at 40 K. Photoemission signals of physisorbed oxygen (α_{u+g}), H_2O (β), O_{chem} (γ) and O_{atomic} (δ) can be identified. Right side: Corresponding O 2s XP spectrum (hυ=150 eV) and the peak assignment.

Pt 4f blank oxidation

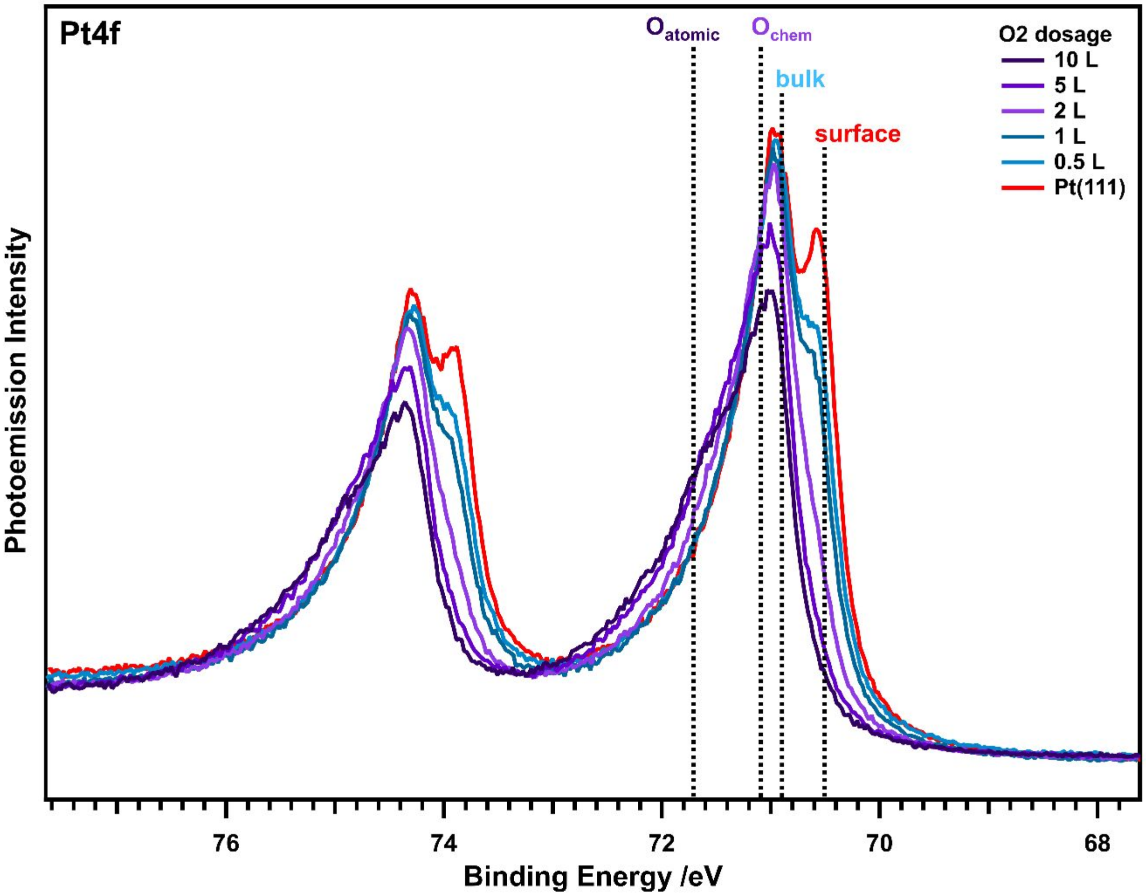

Figure S9. Evolution of the Pt 4f XP spectra (hυ=220 eV) when dosing 0.1-10 L O_2 on Pt(111) at 40 K. The dashed lines and indicated BEs refer to literature values[3,4]. The surface component at 70.5 eV shifts in the presence of oxygen and can no longer be distinguished from the bulk component after exposure to 2 L O_2 and specific O_{chem} (71.1 eV) and O_{atomic} (71.7 eV) surface components appear after dosing >1 L, which are further expressed with higher oxygen exposures. The Pt-bulk component (70.9 eV) is dampened due to surface adsorbates. Considering the low coverage, Ta-related changes in the Pt 4f region are minor, as observed in our previous study, and are expected to display emission features at 71.20 eV[5]. Similarly,

changes with respect to Ta-O-Pt bonds are indiscernible compared to the amount of Pt-O-related contributions.

Table S1. Adsorption energy per Ta atom, calculated Ta 4f binding energy, height with respect to the Pt(111) apical plane, and charges of Ta_4 species on the Pt(111) surface for all atoms (A1-4) in the cluster from top to bottom for both structures respectively. Atom numbers are according to Figure 2(a, b).

Isomer	E_{ads}/atom [eV]	Atom number	BE Ta 4f [eV]	Height [nm]	Q_{Ta} [lel]
$Str_1(Ta_4)$	-3.24	1	23.34	0.21	+1.35
		2	22.93	0.22	+1.32
		3	22.93	0.22	+1.64
		4	23.43	0.18	+1.35
$Str_2(Ta_4)$	-3.96	1	23.80	0.19	+1.35
		2	23.75	0.20	+1.32
		3	23.46	0.01	+1.64
		4	23.80	0.19	+1.35
Ta references					
Ta adatom	-3.84		23.77		
IERTa			23.59		
Ta(110) – rel. to IERTa			22.12		

Table S2. Adsorption energy per Ta atom, calculated Ta 4f binding energy, height with respect to the Pt(111) apical plane, and charges of Ta_8 species on the Pt(111) surface for all atoms (A1-8) in the cluster from top to bottom for both structures respectively. Atom numbers are according to Figure 2(c, d).

Isomer	E_{ads}/atom [eV]	Atom number	BE Ta 4f [eV]	Height [nm]	Q_{Ta} [|e|]
$Str_1(Ta_8)$	-2.13	1	22.72	+0.29	+0.34
		2	23.37	+0.20	+0.93
		3	23.26	+0.18	+0.95
		4	23.13	+0.21	+0.69
		5	22.98	+0.21	+0.72
		6	23.07	+0.21	+0.70
		7	22.95	+0.20	+0.65
		8	23.84	+0.16	+1.17
$Str_2(Ta_8)$	-2.68	1	22.97	+2.19	+1.05
		2	23.31	+2.10	+0.96
		5	23.58	+2.03	+1.11
		4	23.85	+1.79	+1.34
		5	23.58	+2.03	+1.11
		6	22.93	+2.12	+0.80
		7	23.29	+1.89	+1.04
		8	21.98	+3.74	-0.31
Ta references					
Ta adatom	-3.84		23.77		
IERTa			23.59		

Ta(110) – rel. to IERTa	22.12

Table S3. Ta-Ta distances (nm) between all Ta atoms (A1-8) for $str_1(Ta_8)$. Atom numbers are according to Figure 2(c).

Dist (nm)	A1	A2	A3	A4	A5	A6	A7	A8
A1	--	0.348	0.308	0.277	0.273	0.278	0.294	0.599
A2	--	--	0.296	0.517	0.596	0.506	0.276	0.386
A3	--	--		0.290	0.492	0.555	0.462	0.673
A4	--	--			0.279	0.478	0.544	0.837
A5	--	--				0.279	0.481	0.803
A6	--	--					0.280	0.582
A7	--	--						0.323
A8	--	--	--	--	--	--	--	--

Table S4. Ta-Ta distances (nm) between all Ta atoms (A_g1-8) for gas-phase Ta_8, where the index g refers to an atom from a gas-phase structure. Atom numbers are according to Figure S1 (right) for gas-phase Ta_8.

Dist (nm)	A_g1	A_g2	A_g3	A_g4	A_g5	A_g6	A_g7	A_g8
A_g1	--	0.252	0.254	0.252	0.385	0.285	0.286	0.325
A_g2	--	--	0.422	0.300	0.422	0.257	0.427	0.252
A_g3	--	--	--	0.422	0.258	0.261	0.261	0.385
A_g4	--	--	--	--	0.422	0.427	0.257	0.252
A_g5	--	--	--	--	--	0.261	0.261	0.254
A_g6	--	--	--	--	--	--	0.387	0.286
A_g7	--	--	--	--	--	--	--	0.285
A_g8	--	--	--	--	--	--	--	--

Table S5. Adsorption energy per oxygen atom, calculated Ta 4f BE of a Ta adatom in a Pt(111) hcp site and small Ta-oxides TaO_x (x=1-2), Ta-O and Ta-Pt closest interatomic distances (Å).

Structure	E_{ads} [eV]	BE Ta 4f [eV]	R(Ta-O) [Å]	R(Ta-Pt) [Å]
Ta adatom	-3.84	23.77		2.38
TaO	-7.50	22.85	1.75	2.53
TaO_2 (dioxo)	-5.02	22.29	1.87	2.59
Ta_7O		22.92-23.83		2.54-2.59*

*considering oxygen bonded Ta atoms in the cluster

Table S6. Ta-Ta distances (nm) between all Ta atoms (A1-8) for $str_2(Ta_8)$. Atom numbers are according to Figure 2(d).

Dist (nm)	A1	A2	A3	A4	A5	A6	A7	A8
A1	--	0.307	0.408	0.465	0.296	0.291	0.421	0.271
A2	--		0.398	0.680	0.583	0.421	0.296	0.267
A3	--			0.462	0.645	0.647	0.638	0.592
A4	--				0.539	0.727	0.878	0.708
A5	--					0.296	0.575	0.453
A6	--						0.298	0.267
A7	--							0.271
A8	--	--	--	--	--	--	--	--

REFERENCES (SUPPORTING INFORMATION)

(1) Puglia, C.; Nilsson, A.; Hernnäs, B.; Karis, O.; Bennich, P.; Mårtensson, N. Physisorbed, chemisorbed and dissociated O_2 on Pt(111) studied by different core level spectroscopy methods. *Surface Science* **1995**, *342* (1-3), 119–133. DOI: 10.1016/0039-6028(95)00798-9.
(2) Ranke, W. Low temperature adsorption and condensation of O_2, H_2O and NO on Pt(111), studied by core level and valence band photoemission. *Surface Science* **1989**, *209* (1-2), 57–76. DOI: 10.1016/0039-6028(89)90058-7.
(3) Miller, D. J.; Öberg, H.; Kaya, S.; Sanchez Casalongue, H.; Friebel, D.; Anniyev, T.; Ogasawara, H.; Bluhm, H.; Pettersson, L. G. M.; Nilsson, A. Oxidation of Pt(111) under near-ambient conditions. *Physical review letters* **2011**, *107* (19), 195502. DOI: 10.1103/PhysRevLett.107.195502.
(4) Günther, S.; Scheibe, A.; Bluhm, H.; Haevecker, M.; Kleimenov, E.; Knop-Gericke, A.; Schlögl, R.; Imbihl, R. In Situ X-ray Photoelectron Spectroscopy of Catalytic Ammonia Oxidation over a Pt(533) Surface. *J. Phys. Chem. C* **2008**, *112* (39), 15382–15393. DOI: 10.1021/jp803264v.
(5) Bertrang, K.; Hinke, T.; Kaiser, S.; Knechtges, M.; Loi, F.; Sbuelz, L.; Lacovig, P.; Bignardi, L.; Esch, F.; Baraldi, A.; Tosoni, S.; Kartouzian, A.; Heiz, U. Unraveling the interaction of Ta atoms with Pt(111). *Surfaces and Interfaces* **2025**, *56*, 105640. DOI: 10.1016/j.surfin.2024.105640.
